# Emerging Gene Therapeutics for Epidermolysis Bullosa under Development

**DOI:** 10.3390/ijms25042243

**Published:** 2024-02-13

**Authors:** Johannes Bischof, Markus Hierl, Ulrich Koller

**Affiliations:** 1 EB House Austria, Research Program for Molecular Therapy of Genodermatoses, Department of Dermatology and Allergology, University Hospital of the Paracelsus Medical University, 5020 Salzburg, Austria; j.bischof@salk.at (J.B.); m.hierl@salk.at (M.H.); 2 Department of Biosciences and Medical Biology, University of Salzburg, 5020 Salzburg, Austria

**Keywords:** epidermolysis bullosa, gene therapy, gene replacement, gene editing, base editing, prime editing

## Abstract

The monogenetic disease epidermolysis bullosa (EB) is characterised by the formation of extended blisters and lesions on the patient’s skin upon minimal mechanical stress. Causal for this severe condition are genetic mutations in genes, leading to the functional impairment, reduction, or absence of the encoded protein within the skin’s basement membrane zone connecting the epidermis to the underlying dermis. The major burden of affected families justifies the development of long-lasting and curative therapies operating at the genomic level. The landscape of causal therapies for EB is steadily expanding due to recent breakthroughs in the gene therapy field, providing promising outcomes for patients suffering from this severe disease. Currently, two gene therapeutic approaches show promise for EB. The clinically more advanced gene replacement strategy was successfully applied in severe EB forms, leading to a ground-breaking in vivo gene therapy product named beremagene geperpavec (B-VEC) recently approved from the US Food and Drug Administration (FDA). In addition, the continuous innovations in both designer nucleases and gene editing technologies enable the efficient and potentially safe repair of mutations in EB in a potentially permanent manner, inspiring researchers in the field to define and reach new milestones in the therapy of EB.

## 1. Introduction

Epidermolysis bullosa (EB) is a rare, monogenetic skin disease characterised by the formation of extensive blisters and wounds on the skin and mucous membranes upon minimal mechanical trauma. EB shows a broad phenotypic spectrum, ranging from mild to very severe forms associated with a drastically reduced life expectancy [1]. Generally, the disease imposes a substantial burden on the patients and their families [2]. Recent epidemiological studies from England and Wales, as well as the Netherlands, revealed an incidence of 40–60 per 1,000,000 live births and a prevalence of 20–30 per 1,000,000 people for those countries, indicating that EB might be more frequent than previous numbers indicated [3,4,5].

**Figure 1:**
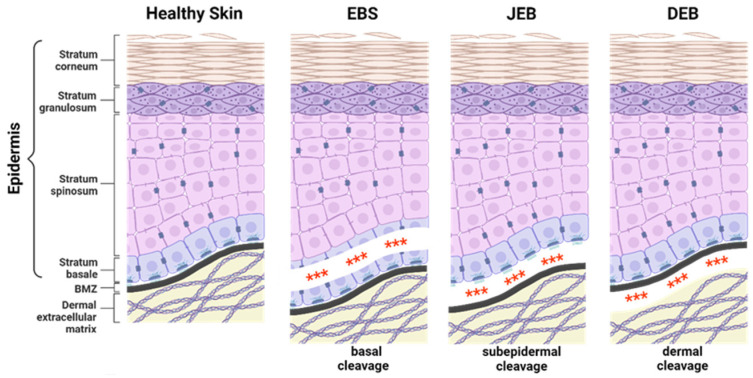
Epidermolysis bullosa subtypes. The three major epidermolysis bullosa (EB) subtypes are defined by the level of tissue separation (***) within the skin. EB simplex (EBS) is characterised by intra-epidermal blistering. In junctional EB (JEB), skin cleavage occurs within the lamina lucida of the basement membrane zone (BMZ). Dystrophic EB (DEB) is caused by the loss of anchoring fibrils leading to skin cleavage directly underneath the lamina densa of the BMZ. The extremely rare Kindler EB (KEB), in which intra-epidermal, junctional, or dermal blister formation can occur. Created with BioRender.com (accessed on 20 December 2023).

EB-causing mutations can be present in at least 16 different genes encoding structural proteins within the skin essential for dermal–epidermal connectivity [1]. The affected gene and the expression level of the corresponding protein contribute to the broad phenotypic variability of the disease. In severe recessively inherited EB forms, the complete absence of the affected protein is associated with more severe disease manifestations compared to EB variants with residual protein expression or impaired protein function [1]. EB can be classified into four major subtypes, defined by the level of tissue separation within the skin (Figure 1). EB simplex (EBS) is frequently caused by mutations in genes coding for keratin 5, keratin 14, or plectin, resulting in intraepidermal blistering. It is commonly inherited in a dominant manner, but recessive cases are also described. EBS is the most common EB subtype [4,5,6] showing a broad phenotypic spectrum, ranging from localised mild to more severe generalised forms [1].

Junctional EB (JEB) is associated with mutations in genes encoding laminin-332, integrin-α6β4, or type XVII collagen (C17) and is recessively inherited. JEB can be divided into generalised severe (formerly known as Herlitz JEB) and intermediate (formerly known as non-Herlitz JEB) JEB. Severe JEB is generally lethal within the first 6 to 24 months after birth [1] and is mainly caused by mutations in *LAMB3* encoding the β-chain of laminin-332 [7]. About 70% of all JEB cases are associated with *LAMB3* mutations [8]. Intermediate JEB, frequently associated with mutations in *COL17A1* encoding type XVII collagen (C17) [1], is characterised by skin cleavage within the lamina lucida of the basement membrane zone (BMZ) between the epidermis and dermis of the skin.

Dystrophic EB (DEB) is associated with mutations in *COL7A1*, encoding type VII collagen (C7), which is expressed by keratinocytes and fibroblasts at the derma-epidermal junction. DEB can be inherited in a recessive (RDEB) or dominant manner (DDEB). Severe RDEB is associated with increased risk of childhood death and a high risk of squamous cell carcinoma (SCC) development in early adulthood [2]. C7 is the major component of anchoring fibrils, which are collagen suprastructures that attach the lamina densa to the underlying dermis. The absence of C7 in DEB impairs anchoring fibril formation, causing skin cleavage directly underneath the lamina densa.

Mutations within *KIND1* are associated with Kindler EB (KEB), an extremely rare recessive EB subtype, in which intraepidermal, junctional, or dermal blister formation can occur [9]. *KIND1* encodes kindlin-1, an intracellular protein involved in focal adhesion.

The disease burden for EB patients, and in particular of those suffering from more severe EB forms, underlines the need for effective treatment options. However, up to this point, the treatment of EB patients is still mostly restricted to wound management, as no permanent cure for EB is available [10]. Gene therapy appears to be the only treatment approach to potentially cure the disease. This review gives an overview of gene therapeutics under development for future clinical applications in EB. A special focus is dedicated to gene editing, which has not reached the clinic yet. However, the scientific community in EB currently consider gene editing as the most promising treatment approach with the potential to cure EB in the future.

## 2. EB Therapeutics under Development

### 2.1. Autologous and Allogenic Cell Therapies for EB

In a pioneering study, Carter et al. repeatedly transplanted autologous epidermal grafts on the chronic wounds of three JEB patients. The grafts were generated with keratinocytes harvested from non-wounded skin areas grown on collagen sponges. This strategy closely resembles the autologous graft delivery performed later in ex vivo gene therapy, which is discussed in detail below. Two of the three JEB patients showed complete re-epithelisation of the treated wounds 7 and 10 months after treatment, indicating improved wound healing [11]. However, even though the wounds were less severe, the cause of the underlying disease was not reversed [12].

Revertant mosaicism, a phenomenon where a disease-causing mutation is corrected naturally by a somatic change, represents a source of autologous cells with potential therapeutic usability. In the context of EB, revertant mosaicism leads to small patches of wild-type skin on EB patients. It was initially confirmed for a JEB patient with compound heterozygous *COL17A1* mutations [13]. At first considered a rare phenomenon, revertant mosaicism was, in a later study, shown to be present in the majority of patients with *COL17A1*-associated JEB; it has subsequently been detected in all major EB subtypes [14,15]. Although a proof of principle transplantation of autologous revertant keratinocytes was successfully performed on a laminin-deficient patient, several obstacles, including the isolation of revertant stem cells, need to be addressed before it can provide a safe and efficient therapeutic treatment option [15,16].

In contrast to autologous approaches, various allogenic cell therapies were explored in clinical trials [17]. Allogenic engineered skin grafts (graftskins), which were originally approved for the treatment of venous ulcers, were tested in uncontrolled clinical studies published in the early 2000s [18,19]. Graftskins are generated from neonatal keratinocytes and fibroblasts grown on a bovine collagen matrix [20]. Patients with different EB subtypes participating in these studies reported improved wound healing efficacy and reduced pain compared to conventional wound treatment. However, the beneficial effects were only short-term.

Other studies took different approaches, such as the injection of allogenic fibroblasts into the wounds of RDEB patients. Wounds treated this way showed significantly improved wound healing compared to untreated wounds in two clinical trials [21,22]. Unfortunately, the improved wound healing only lasted one month following the allogenic fibroblast injection [21]. Surprisingly, the vehicle treatment showed improved wound healing compared to untreated wounds and no significant difference in healing between the vehicle-treated and actually treated wounds was observed [22]. The transplantation of allogenic mesenchymal stem cells (MSCs), cells with the capacity to develop into C7-expressing fibroblasts, was evaluated in several RDEB clinical trials. MSCs were injected locally [23] or systemically [24,25], leading to increased dermal–epidermal stability and detectable C7 expression up to 4 months after treatment.

Bone marrow transplantation (BMT) has the potential for longer persistence of allogenic cells in recipient patients, possibly providing a significant benefit compared to the allogenic strategies discussed above [17,26]. In addition, mucous areas, which are difficult to target, could benefit from this strategy. Pre-clinical mouse data showed promising results for BMT as a potential treatment option for RDEB. *Col7a1*^−/−^ pubs develop a severe RDEB phenotype leading to death within the first two weeks of life [27]. Intravenous injection of CD150^+^/48^−^-enriched bone-marrow-derived cells into *Col7a1*^−/−^ pubs resulted in a 15% long-term survival rate when monitored for 10 weeks post treatment. Injected cells migrated to RDEB wounds and induced C7 expression, resulting in the partial formation of anchoring fibrils [28].

The first BMT-based clinical trial for RDEB patients yielded improved wound healing and reduced blister formation with C7 deposition within the dermal–epidermal junction between 30 and 130 days after BMT, albeit no anchoring fibril formation was detected. The treatment had severe side effects. Two of the seven participating patients died over the course of the clinical study. The first patient died during preparation for the transplantation due to a cardiomyopathy likely caused by cyclophosphamide. The second patient passed away 183 days after transplantation due to infections associated with graft rejection [26].

The first JEB patient who received allogenic stem cells, a newborn with severe JEB, also suffered from multiple viral and bacterial infections after treatment, highlighting the severe risk of BMTs for EB patients [29,30]. A second laminin-332-deficient newborn, who also received a BMT, suffered from severe side effects due to the treatment. Up to 7 weeks after BMT, the patient showed an improved skin status and stable body weight. However, while skin biopsies showed the presence of donor cells, laminin-332 was not detected. Skin stability and body weight started to decline 7 weeks after BMT. The baby died 129 days after transplantation due to a severe infection [30].

### 2.2. Splicing Modulation via Antisense Oligonucleotides

Antisense oligonucleotides (ASO) are commonly used to modulate splicing in genetic diseases such as EB. As an emerging therapeutic platform, ASOs can be exploited to influence various aspects of (pre-)mRNA biology, including splicing, stability, and translation. ASOs can bind to mutated in-frame exons during pre-mRNA splicing in order to induce exon skipping [31], or can be used to enhance accurate splicing via modulating the general splicing pattern in the target cells [32]. Exon skipping leads to truncated proteins, which could have a negative impact on protein function. This should be considered in preclinical studies already performed for *COL7A1* [33,34,35] and *COL17A1* [36] in EB. Turczynski et al. successfully demonstrated exon 70/80 skipping in RDEB fibroblasts and keratinocytes via 2′-MOE ASOs to restore C7 expression and anchor fibril formation [35]. For exon 73, a phase I/II clinical trial, based on ASO(QR-313)-mediated exon skipping [34], was initiated by Wings Therapeutics. Unfortunately, the clinical study was recently terminated due to low enrolment (NCT03605069). As opposed to the exon-skipping strategy, Hainzl et al. recently applied an exon-inclusion approach for RDEB [32]. Here, a disease-associated splice site mutation (c.425A>G) in *COL7A1* reduced the efficiency of correct splicing at the exon/intron junction, resulting in mainly non-functional C7 transcripts. The performance of a target locus-specific 2′-MOE ASO screen led to the identification of a lead ASO capable of increasing the relative levels of accurately-spliced *COL7A1* transcripts and consequently “full-length” C7. Furthermore, an accumulation of “full-length” C7 was detectable at the basement membrane zone of ASO-treated skin equivalents. In summary, ASO-mediated splice modulation is a promising option for the treatment of EB, although challenges related to ASO delivery into the skin must be solved prior to a potential clinical application.

### 2.3. Gene Therapies for EB

**Figure 2:**
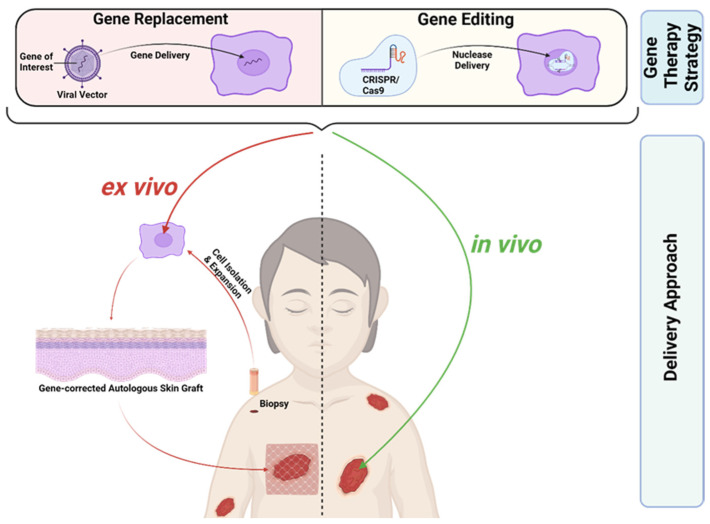
Gene Therapies for EB. Gene replacement therapies rely on viral-vector-based gene delivery, in contrast to gene editing therapies, where a nuclease is delivered to specifically target a mutated gene locus. CRISPR/Cas9 represents the most advanced gene editing tool today. Gene therapies can be applied ex vivo or in vivo. In ex vivo gene therapies the patient’s skin cells are isolated from skin biopsies and subsequently treated in vitro in order to induce re-expression of the corrected protein. The gene-corrected cells are then used for the generation of autologous skin grafts, which are subsequently transplanted back onto the patient. In contrast, in vivo gene therapy is based on the direct local treatment of the patient’s skin. Created with BioRender.com (accessed on 20 December 2023).

EB, as a severe monogenetic skin disease, represents a suitable target for gene therapy. Gene therapeutic approaches can focus on gene disruption, gene replacement, and gene editing [37]. RDEB and JEB, the two most severe EB subtypes, are caused by recessive mutations. Here, gene replacement and gene editing represent potential treatment options. Gene replacement is clinically more advanced compared to gene editing and has been successfully applied to patients in clinical trials, whereas gene editing is still at a preclinical stage. Most gene therapeutics for EB are applied ex vivo (Figure 2). Here, a patient’s skin cells are isolated from skin biopsies and subsequently treated in vitro in order to induce the re-expression of the corrected protein. The treated, gene-corrected cells are then used for in vitro generation of autologous skin grafts, which are subsequently transplanted back onto the patient. An alternative therapeutic strategy is based on the local treatment of the patient’s skin in vivo (Figure 2). Recently, the first in vivo treatment option for EB based on gene replacement was approved by the FDA, giving hope to patients suffering from EB [38].

An overview of all completed, ongoing, and terminated gene therapy clinical trials for EB are listed in Table 1. To date, all EB-related clinical trials are exclusively based on gene replacement.

### 2.4. Ex Vivo Gene Replacement Clinical Trials

The first gene replacement clinical trial was carried out on a 36-year-old JEB patient with non-lethal double-heterozygous mutations in *LAMB3*. *LAMB3* encodes the β3 chain of laminin-332. An ex vivo gene replacement strategy was applied in this phase I/II clinical trial. Primary patient keratinocytes were obtained from skin biopsies and subsequently transduced with a Moloney leukaemia virus (MLV)-derived retroviral vector expressing full-length *LAMB3* cDNA. Transduction efficiency was close to 100%; autologous epidermal grafts generated with these corrected keratinocytes were subsequently transplanted onto the patient, resulting in improved skin integrity in the treated areas [39]. The engrafted areas showed normal laminin-332 expression levels and correct laminin-332 deposition at the dermal–epidermal junction (DEJ) 4 months after transplantation. In a long-time follow-up 6.5 years after transplantation, the engrafted areas showed normal laminin-332 expression and blister-free skin. In a follow-up 16 years after transplantation, the transgenic skin proved to be stable, indicating a permanent clinical outcome [42,43,44]. In addition, no tumour development or other severe adverse events were observed during the 16-year follow-up [42].

In recent years, two other intermediate JEB patients with *LAMB3* mutations were successfully treated using the same ex vivo approach. In a single case study, a 49-year-old woman received two transgenic autologous epidermal grafts on an approximately 80 cm^2^ wound. The transplantation led to stable skin, confirmed laminin-332 expression and the formation of hemidesmosomes, highlighting the potential to treat large chronic wounds via a combined ex vivo cell and gene therapy in JEB. In a one-year follow-up, normal laminin-332 expression levels and correct protein deposition within the BMZ were confirmed [41]. In a life-saving phase I/II clinical trial, a 7-year-old boy with a homozygous *LAMB3* acceptor splice site mutation was treated. The boy suffered from a severe infection, leading to epidermal loss at about 80% of his total body surface area. Numerous autologous transgenic epidermal grafts, with a total size of about 0.85 m^2^, were transplanted on the boy’s body surface. This intervention led to a stable skin with normal laminin-332 expression levels and correctly localised laminin-332 on the entire transplanted area [40]. In addition to this life-saving intervention, Hirsch et al. analysed the integration profile of the transgenic epidermis, which provided evidence that the human epidermis is sustained by a small number of stem cells termed holoclones [45]. This insight highlights the importance of holoclone targeting for long-term therapeutic success of gene therapies for skin disorders. In a five-year follow-up, the boy’s transgenic epidermis was stable with normal laminin-332 expression and correct laminin-332 deposition. In addition, Langerhans and Merkel cells, as well as sebaceous and sweat glands, were present in the restored skin [46]. Thermoreception and nociception was within, or close to, the normal range and no adverse events were observed 5 years after transplantation. Although the mechanical reception was below the normal range, and a mild form of fibrosis was observed 5 years after transplantation, this is clearly an outstanding study [46].

The long-term safety and success of the combined ex vivo cell and gene therapy treatment of JEB patients initiated an ongoing phase II/III clinical trial to prove the efficacy and safety of this strategy (NCT05111600). The clinical trial started in 2022, involves about six patients and is estimated to be completed mid-2024.

A similar gene replacement strategy was recently applied for RDEB in a phase I clinical trial. Siprashvili et al. [47] described the first combined ex vivo cell and gene therapeutic application for RDEB (NCT01263379) targeting the wounds of four C7-deficient patients. Patient keratinocytes, obtained from skin biopsies, were transduced with the MLV-derived retroviral vector LZRSE-*COL7A1* for full-length *COL7A1* cDNA delivery. A total of 24 grafts were transplanted onto 6 wounds per patient, of which 19 were chronic, present for at least 3 years, with a majority of wounds present for more than 5 years. No serious adverse events were observed in this open-label trial. Three months after transplantation, wound healing displayed major improvements at 21 of 24 graft sites, showing healing of ≥75%. Furthermore, 90% of biopsy samples from grafted areas showed C7 expression, in contrast to untreated patient skin samples, as assessed by immunofluorescence microscopy. Moreover, C7 was accurately localised at the DEJ and anchoring fibril formation was observed. However, C7 expression in skin biopsy samples taken from the graft sites declined from 90% to 42% within the first year after transplantation. Correspondingly, the beneficial effects on wound healing declined within this time frame [47].

In a later phase I/IIa RDEB clinical trial involving seven patients, the same ex vivo gene replacement strategy was applied (also listed as NCT01263379). This controlled open-label trial, which included a long-term follow-up of the four patients participating in the phase I RDEB trial mentioned above, reported significantly improved wound healing over 2 years after transplantation [48]. The majority of treated wounds remained at least 50% healed 2 and 3 years after transplantation. Two patients had C7 expression for up to 2 years after transplantation and no severe adverse events were observed. However, also in this study, a decline in C7 expression, associated with loss of the aforementioned beneficial effect on wound closure and wound healing, was evident [48]. A 5-year follow-up of all seven treated patients confirmed the strategy’s safety and improved wound healing. In addition, significantly reduced itch and pain was reported by patients. Nevertheless, wound healing of 50% or more was declining within the 5-year follow-up, from 93% of grafted sites after 6 months to 70% of the grafted sites at year 5 [49]. A Phase III controlled trial to further evaluate the safety and efficacy of LZRSE-*COL7A1* engineered autologous grafts was completed at the end of 2022 (NCT04227106). Unfortunately, no clinical data have been published yet. Recently, an open-label uncontrolled phase IIIb RDEB clinical trial was started to further evaluate the safety of this retroviral gene replacement strategy (NCT05725018). The trial involves 10–12 patients and is estimated to be completed by the end of 2024.

In contrast to the ex vivo gene therapy in JEB, where laminin-332 was stably expressed more than a decade after transplantation, C7 expression declined within the first year after the transplantation of gene-corrected grafts onto RDEB patients [47]. One reason for the observed decline might be limited LZRSE-*COL7A1* targeting of epidermal stem cells, since these cells are responsible for sustaining and renewing the human epidermis [17,40]. Another reason might be the lack of a selection advantage of corrected C7-expressing RDEB cells compared to non-corrected cells. This contrasts with the JEB-associated ex vivo gene therapies discussed above, where laminin-332-expressing keratinocytes display an adhesive advantage over uncorrected laminin-332-lacking keratinocytes in vitro as well as in vivo [50,51].

The intradermal injection of genetically corrected C7-expressing autologous fibroblasts is a less invasive ex vivo approach compared to transplantations since surgeries are not required. An ex vivo fibroblast-based gene replacement strategy was first applied in a human RDEB xenograft mouse model [52]. Here, fibroblasts were transduced with a self-inactivating retroviral vector. Correct C7 deposition and anchoring fibril formation at the DEJ after injection of the gene-corrected fibroblasts was shown. Motivated by these promising preclinical results, a phase I and a phase I/II clinical trial were started (NCT02493816, NCT02810951) [53,54]. In both trials, a self-inactivating lentiviral vector was used for *COL7A1* cDNA delivery into autologous fibroblasts. Injection of the gene-corrected fibroblasts into defined skin areas was generally well tolerated by patients in both studies. Furthermore, C7 was correctly localised at the DEJ and improved wound healing was reported after treatment. However, in one of the trials (NCT02493816), C7 expression levels remained rather low in some patients. Additionally, none of the treated areas showed the formation of anchoring fibrils in the first 12 months of monitoring [53]. In contrast to this study, a second study (NCT02810951) revealed anchoring fibril formation in at least one patient 25 weeks after the injection of gene-corrected fibroblasts [54]. An intra-patient, randomised, controlled, open-label phase 3 clinical trial involving six RDEB patients is currently being carried out (NCT04213261) to further evaluate the efficacy of this strategy.

### 2.5. In Vivo Gene Replacement Clinical Trials

C7 decline after transplantations of gene-corrected autologous grafts for RDEB treatment, as discussed above, might indicate that periodic or at least repeated application of a gene therapy agent might be required to achieve the best clinical benefit, at least in RDEB [47,55]. Combined ex vivo cell and gene therapy is accompanied by hospitalisation, general anaesthesia, invasive surgery, and subsequent immobilisation, at least in cases of graft transplantation [56]. It holds a high interventional burden and repeated application might not be feasible or desired from the patient’s side. Additionally, resource-intensive cell expansion at specialised manufacturing centres, as well as biopsy collection, is required, which is also true for the application of gene-corrected fibroblast injections [40,41,47,53]. In vivo gene therapy treatments, where a therapeutic agent is directly applied to patient skin, reduces the interventional burden for EB treatment compared to ex vivo strategies (Figure 2).

In 2023, the first gene therapy pharmaceutical for EB was approved by the FDA: Vyjuvek™ by Krystal Biotech [57]. Vyjuvek™ represents an in vivo gene replacement gene therapy for the topical treatment of RDEB wounds. Two ground-breaking clinical trials preceded the FDA approval of Vyjuvek™ (NCT03536143, NCT04491604) [55,58]. Here, gene delivery was enabled by an engineered, non-replicative herpes simplex virus type 1 (HSV-1), which contains two full-length copies of *COL7A1* cDNA, called beremagene geperpavec (B-VEC). HSV-1 has natural immune-evasive properties, thereby reducing vector-induced immune reactions. Preclinical data showed that B-VEC restored full-length C7 expression if topically applied to wounds of C7-deficient mice. The same effect was observed when B-VEC was applied to human xenografts on immunodeficient mice. Furthermore, anchoring fibrils were detected in biopsies of B-VEC-treated human xenografts, indicating that B-VEC induced functional C7 expression in human xenografts.

The following randomised, placebo-controlled phase I/II clinical trial (NCT03536143) enrolled nine RDEB patients, who received repeated topical B-VEC administration over 3 months [55]. The treatment was well tolerated, and no serious adverse events were observed. One moderate adverse event was reported but was most likely not related to the B-VEC treatment. In total, 18 wounds were repeatedly treated with B-VEC, of which 17 were completely closed after the 3-month treatment time. The 10 placebo-treated wounds fluctuated between healing and re-blistering, a typical behaviour of RDEB wounds. The baseline wound size of the treated wounds was between 0.59 and 65.29 cm^2^. Complete wound closure was significantly improved in the treatment group after 3 months. The mean percentage change in the wound surface area compared to the baseline wound surface area was significantly improved in the B-VEC treatment group 8, 10, and 12 weeks after treatment. The median time to complete wound closure was 13.5 days for the treatment group, compared to 22.5 days for the control group. Mean duration of closure was 103 days for the B-VEC treatment group, in contrast to 16.5 days for the placebo-treated group. C7 NC1 and NC2 domain expression was observed via immunofluorescence microscopy in most post-treatment biopsies, showing full-length C7 expression correctly located at the DEJ. NC1 and NC2 domain expression, along with anchoring fibril formation, was confirmed in all samples analysed via immunoelectron microscopy [55].

Due to these encouraging results, B-VEC’s efficacy and safety were further evaluated in a randomised, placebo-controlled phase III clinical trial (NCT04491604) [58]. Here, primary wound pairs matching in size, region, and appearance were determined for each of the 31 participating patients, accomplishing an intrapatient control design, as suggested by the FDA. One wound of each pair was treated weekly for 6 months with topologically applied B-VEC, the other wound received placebo treatment. The treated wound areas ranged from 2.3 to 57.3 cm^2^, with a median wound area of 10.6 cm^2^, very similar to the placebo-treated wounds. B-VEC and placebo treatments were only applied if wounds were not closed. Complete wound closure (defined as 100% wound closure for at least 2 weeks, as suggested by the FDA) was significantly enhanced in the B-VEC treatment group compared to the placebo group at 3 as well as at 6 months after treatment; 67% of wounds treated with B-VEC were completely closed after 6 months compared to 22% of the placebo-treated wounds. After 3 months of the treatment, 71% of the B-VEC-treated wounds were completely closed, compared to 20% in the placebo group. Larger wounds were treated with B-VEC and qualitatively observed. The almost-complete closure of a chronic wound with >100 cm^2^ after B-VEC treatment is a striking example of B-VEC’s efficacy in large wounds. Regarding safety, five serious adverse events were documented over the course of the 6-month trial, which were all deemed to be not related to either the B-VEC or the placebo treatment. Mild erythema, likely related to the B-VEC treatment was observed in one patient. Pruritus and chills occurred in three patients after the B-VEC treatment. No immunologic reaction with clinical significance was documented, allowing for reiterated B-VEC application [58]. An observational, long-term follow-up study, involving all patients treated with a HSV-1 vector (including non-EB patients), is ongoing and estimated to be completed in 2028 (NCT04917887).

Following the phase III clinical trial, the FDA approved B-VEC under the name Vyjuvek™ for the topical treatment of RDEB wounds. Vyjuvek™ represents not only the first FDA-approved gene therapy drug for EB treatment, but also the first ever FDA-approved topical gene therapy [57]. This trailblazing accomplishment theoretically enables direct in vivo gene therapy in all RDEB patients. B-VEC in vivo gene therapy shows many advantages compared to ex vivo strategies. Patients are no longer required to travel to hospitals specialising in EB treatment, which is not always affordable for many EB patients; biopsy, hospitalisation, anaesthesia, or surgery followed by immobilisation is not required. In contrast to other in vivo gene therapies, immunosuppression is not required for B-VEC, due its immune-evasive properties. Compared to ex vivo gene therapy, which relies heavily on retroviral vectors, safety is increased, since the non-integrative nature of B-VEC avoids insertional mutagenesis [55,59].

Despite all the advantages, B-VEC has limitations. The major constraint of B-VEC is its incapacity to penetrate intact skin, which restricts it to the treatment of RDEB wounds. Wound prevention is not feasible with Vyjuvek™. Repeated application of a gene therapy agent, essential for B-VEC’s clinical benefit, is expensive. Krystal Biotech estimated the costs as US$ 631,000 per patient annually [57]. At this price, the treatment will not be affordable for all patients. A potential future in vivo gene editing approach, where a one-time treatment could lead to correction of the disease-associated mutation, might be less expensive and therefore reach more patients.

### 2.6. Gene Editing in Clinical Use

In contrast to gene replacement strategies, gene editing for EB is still at a pre-clinical stage. A stark difference between the two approaches is that replacement therapies rely heavily on viral-vector-based gene delivery (Figure 2) [10]. Retroviral vectors, which are used in ex vivo gene therapy clinical trials for EB, permanently integrate into the genome [39,40,41,47]. Retroviral integration, a semi-random process in which the integration site cannot be controlled, is potentially carcinogenic due to insertional mutagenesis. This was, in fact, observed in a limited number of patients who developed leukaemia after treatment with a retrovirus-based strategy for X-linked severe combined immunodeficiency and chronic granulomatosis disease [60,61]. However, thus far, most EB patients treated with an ex vivo gene replacement approach tolerated the treatment well and no carcinogenesis was observed [43,46,49]. Nonetheless, the FDA suggests monitoring patients treated with integrating vectors for at least 15 years due to potential delayed adverse events [62]. In contrast to replacement therapies, gene editing approaches specifically target the mutated gene locus (Figure 2). They enable permanent repair of mutations with restored endogenous gene expression and without reliance on viral vectors [10].

Designer nucleases represent the most advanced gene editing tool today. They are composed of two parts, a programmable DNA-binding domain, and a nuclease [63]. Following DNA binding, a DNA double-strand break (DSB) is introduced and subsequently repaired by cellular repair pathways. The most prominent DSB repair pathways are non-homologous end-joining (NHEJ) and homology directed repair (HDR), typically resulting in the formation of small insertions or deletions (indels) and perfect repair outcomes, respectively. NHEJ, which is active throughout the cell cycle, is suitable for disruption and re-framing of genes [64,65,66]. In contrast to NHEJ, HDR requires a DNA donor template allowing for the substitution of a specific sequence or the introduction of larger DNA sections. Providing an exogenous template ultimately enables traceless gene editing. At the same time, HDR, which is only active in the late G1- and S-Phases of the cell cycle, shows reduced efficiency compared to NHEJ [63].

In recent years, CRISPR/Cas9 has emerged as the most versatile tool in genome editing. This is due to the fact that it is a very robust and easy-to-use system. In fact, it only requires two components to work: the Cas9 protein with its DNA cleavage domains and the sgRNA, responsible for guiding the nuclease to the target locus of interest. The sgRNA can be further divided into crRNA and tracrRNA [67]. The CRISPR/Cas9 system is delivered as a plasmid, mRNA, or as ribonucleoprotein (RNP). For a comprehensive review of EB-related, classical CRISPR/Cas9-based gene editing experiments, please refer to Kocher et al. [10].

In addition to Cas9, other CRISPR/Cas-systems also hold huge potential for gene editing purposes. Among those, Cas12, another class 2 system, was extensively studied in the last few years, especially Cas12a (Cpf1) [68]. In contrast to Cas9, Cas12a only requires the crRNA for efficient targeting [69]. It recognizes T-rich PAM regions (5’-TTTV-3’) and is therefore well suited for the use at AT-rich regions [69,70,71]. In addition, Cas12a produces staggered cuts, possibly enhancing HDR frequencies when compared to Cas9 [72,73,74]. Other features include high specificity and the small size of Cas12a [75,76]. Although not yet as far advanced as Cas9 in terms of clinical studies, Cas12a has been used in multiple gene editing experiments over the course of the last decade [77].

OMEGA (obligate mobile element guided activity) is another RNA-guided endonuclease system. Its effectors, TnpB and Fanzor, are found in prokaryotes and eukaryotes, respectively. Both are components of the transposable element and contain, similar to Cas12, an RuvC domain. However, they are significantly smaller in size [78,79,80]. Indeed, TnpB is thought to be the ancestor of Fanzor and the Cas12 system [78]. In contrast to Cas12, they are not associated with an sgRNA but a so-called ωRNA. For Fanzor endonucleases, the 3’-terminal ωRNA flanking sequence was proposed to be responsible for targeting. In addition, most Fanzor endonucleases were found to create staggered cuts. Even though gene editing efficiencies have been relatively low so far, the OMEGA endonuclease systems are certainly worth pursuing and improving [80].

In terms of CRISPR/Cas9-based technologies, several new variants have evolved in recent years, with the most prominent being base editing (BE) and prime editing (PE). In BE, a deaminase is fused to Cas9. It is suitable for performing all four transition point mutations (C → T, T → C, A → G, G → A) without creating a DSB [81]. PE is a technology where a reverse transcriptase is fused to Cas9. Theoretically, PE allows for the insertion of any desired edit at a targeted locus [82].

In contrast to EB and genodermatoses in general, where no gene editing therapy has reached a clinical application so far, CRISPR/Cas9 systems are employed in clinical trials for various other diseases. In late 2023, more than 50 clinical trials with CRISPR/Cas9-based therapeutic agents were listed on the clinicaltrials.gov (accessed on 15 December 2023) database. This makes CRISPR/Cas9 by far the most commonly applied gene editing tool in clinical trials today. In these trials, CRISPR/Cas9 is most frequently used to treat blood disorders, malignant diseases, and metabolic disorders (https://clinicaltrials.gov/, accessed on 15 December 2023).

The first CRISPR/Cas9-based clinical trial started in 2016 for the treatment of non-small cell lung cancer [83]. Currently, several companies are funding ongoing clinical trials to treat hemoglobinopathies like β-thalassemias and sickle cell disease. Hemoglobinopathies are the most frequent monogenetic disorders, which cause more than 3% of deaths in children younger than 5 years [84]. The clinically most advanced gene editing strategy aims to restore expression of foetal haemoglobin (HbF), which is, in general, silenced 3 months after birth. Restored HbF compensates for the absence of fully functional haemoglobin in hemoglobinopathies. CRISPR/Cas9 is used to either disrupt HbF repressors or for the editing of HbF regulatory elements to prevent repressor binding [85,86]. The most clinically advanced is CTX001, an autologous, ex vivo CRISPR/Cas9 gene-editing therapy, which is currently being evaluated in several phase III clinical trials. CTX001 disrupts an enhancer region of BCL11A, a HbF repressor, thereby increasing HbF [86]. According to Vertex Pharmaceuticals (Boston, MA, USA) and CRISPR Therapeutics (Zug, Switzerland), the sponsors of the trial, this strategy proved to be successful. 42 of 44 treated sickle cell patients were no longer transfusion-dependent [84].

Although most CRISPR/Cas9-based clinical trials are ex vivo approaches, in 2019 the first in vivo CRISPR/Cas9-based gene editing agent, EDIT-101, reached the clinic. The safety and efficacy of EDIT-101 for the treatment of Leber congenital amaurosis are being evaluated in a phase I/II clinical trial, estimated to be completed in 2025 [83]. In addition, a next-generation CRISPR/Cas9-based editing technology, base editing, is already in clinical use, which is discussed in more detail below. It will be clinically evaluated in currently recruiting phase I/II clinical trials for the treatment of hemoglobinopathies and cancer (https://crisprmedicinenews.com/clinical-trials/ (accessed on 11 November 2023)).

## 3. Efficiency and Specificity of CRISPR/Cas9

In recent years, the safety and specificity of CRISPR/Cas9 has steadily improved. The development of in silico off-target prediction tools as well as off-target cleavage detection assays has become a main focus in the research field, making CRISPR/Cas9-based clinical studies a reality. Cas9-mediated cleavage at off-target sites, highly homologous to the sgRNA-binding site, can induce DNA damage with serious consequences for the cell [87]. Therefore, it is important to carefully develop gene editing strategies, considering sgRNA design and Cas9 enzyme selection.

Essential for DNA targeting and its subsequent cleavage is the 20-nucleotide-long spacer region of the crRNA. Most important are the first five nucleotides adjacent to the protospacer adjacent motif (PAM) (PAM-proximal or seed region). Mismatches as well as DNA/RNA bulges can sometimes be tolerated, although binding at an off-target site does not automatically lead to DNA cleavage. Even though the crRNA is responsible for targeting DNA, the tracrRNA can also influence editing efficiency. It impacts the stability of the whole RNP complex by contributing to Cas9-enzyme binding [88,89,90,91,92,93]. The Cas9 protein itself is activated by sgRNA-binding and promotes, upon PAM recognition, local DNA melting, allowing for the sgRNA to base-pair with the target DNA. Cas9 enzymes can also be modified to generate protein variants capable of cleaving only one DNA strand [94,95,96]. The specificity and efficiency of the CRISPR/Cas9 system can be increased by modifying the sgRNA components and/or the Cas9 enzyme. However, modifications of these two components require excessive screening procedures, since they could also lead to decreased on-target, and/or increased off-target activity.

### 3.1. Modulation of sgRNA Efficiency via Chemical Modifications, Truncations or Extensions

The sgRNA contains a 36–43-nt-long crRNA and a tracrRNA of 67–89 nt in length. Both RNA segments anneal to each other at repeat and anti-repeat regions to form the sgRNA molecule [67,97,98]. sgRNA synthesis has been simplified by covalently linking a 20-nucleotide crRNA with a 79-nucleotide tracrRNA, making the procedure more cost-effective, thereby enabling quick formational changes.. At the same time, the editing efficiency of these modified sgRNAs remained the same [99,100]. However, in addition to efforts to streamline sgRNA synthesis, modifications of the sgRNA were performed to improve efficiency and/or specificity. An important issue regarding sgRNA stability and, consequently, effectiveness, is the fact that they can be degraded by intracellular endo- and exonucleases [101]. In order to make them more stable, it is possible to chemically modify their termini or internal residues. In addition, truncations/extensions and RNA substitutions of sgRNA residues can also impact their specificity. For an overview, please refer to Table 2.

### 3.2. Cas9 Variants and Their Impact on Editing Specificity

The CRISPR/Cas9 system is an RNA-guided endonuclease. Its initial purpose in bacteria is to provide adaptive immunity against foreign genetic elements. The CRISPR/Cas9-locus found in bacteria consists of sequences encoding the Cas9 protein and a repeat-spacer region. As mentioned before, the repeat-spacer region encodes the crRNA responsible for guiding the attached Cas9 protein to the target locus. The Cas9 enzyme facilitates the cleavage of the DNA single or double strand [67,116,117,118]. The widely used SpCas9 protein is a multidomain protein with a size of 1386 aa. It consists of nuclease (NUC) and recognition (REC) lobes connected by a bridge helix (BH). The two endonuclease domains, HNH and RuvC, responsible for creating a double-strand break, are found in the NUC lobe. While the HNH domain cleaves the target strand, the RuvC domain cleaves the non-target strand. The PAM-interacting (PI) domain is also found in the NUC lobe. The REC lobe, on the other hand, is responsible for the binding of Cas9 to tracrRNA and the (target) DNA [94,95,119,120,121,122,123].

**Figure 3:**
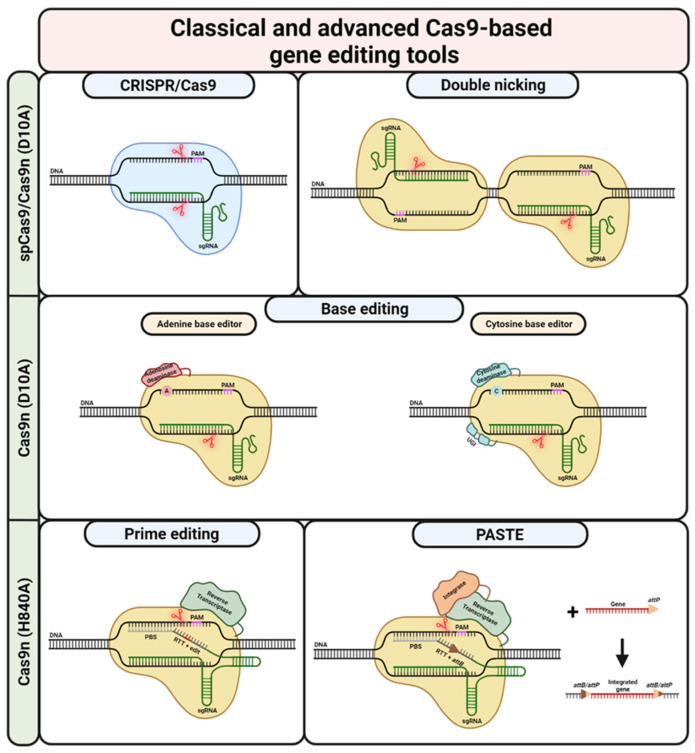
CRISPR/Cas9-based gene editing tools. Classical CRISPR/Cas9-based editing is based on DSBs (DNA cleavage site(s) indicated via red scissors) created by spCas9. The cell’s own repair machinery creates indels, allowing for gene knock-out or -reframing. By providing a repair template, the outcome can by directed towards the HDR pathway, ultimately leading to traceless correction. Repair templates can also be combined with Cas9n, avoiding possibly adverse off-target effects arising from the creation of DSBs. Double nicking is another possibility for using Cas9n. In this case, in order to create a (staggered) DSB, it is necessary to use two sgRNAs binding and cleaving in close proximity on opposite strands. Similar to the default CRISPR/Cas9-approach, DN can be used for gene knockout and gene reframing as well as HDR. Base editing is possible via the fusion of Cas9n (initially dCas9) to adenosine/cytosine deaminases. While adenosine deaminases allow for the conversion of adenosine to guanosine, cytosine deaminases are able to convert cytidine to thymidine. The nick created by the Cas9n (D10A) mutant recruits the cell’s repair machinery, ultimately increasing the chances of installing the desired edit on the second DNA strand. In contrast to base editing, prime editing utilizes the Cas9n (H840A) mutant fused to a reverse transcriptase. PE uses a pegRNA, combining a default sgRNA with 3’ extensions consisting of PBS and RTT. While the PBS binds to the DNA strand initially untargeted by the spacer/sgRNA 3’ of the nick, the RTT provides a template for the RT and includes the desired edit. This edit can vary in length and composition, making PE a hugely versatile gene editing tool. If the newly RT-synthesised strand is incorporated, the edit is successfully installed on one DNA strand. In order to install the edit on the second strand, a so-called nicking sgRNA can be used. Similar to base editing, the nicking of the unedited strand recruits the cellular repair machinery. This machinery then uses the edited strand as a template for the repair. Prime editing is also the basis for PASTE. However, PASTE uses the PE reaction only to install an Bxb1 integrase attB site at a desired locus. The Cas9n (H840A) mutant is fused not only to a RT but also to a Bxb1 integrase. Once the attB site is in place, a gene or sequence of interest with an attP site can be incorporated at this site. This theoretically allows for the integration of sequences with up to several kilobases. UGI = uracil glycosylase inhibitor. Created with BioRender.com (accessed on 20 December 2023).

Soon after it was recognised that the CRISPR/Cas9 system can be used as a gene editing tool, the scientific community started working on modifying the Cas9 enzyme (Figure 3). The intention was to increase the specificity, i.e., increase on-target and decrease off-target binding/cleavage. Several groups have worked on destabilizing the RNP/DNA complex as well as on modifying the REC lobe to achieve less efficient conformational changes between the active and inactive states of the protein. This was predominantly achieved by the mutagenesis of amino acids involved with binding the targeted and non-targeted DNA strand. In addition to the REC lobe, the Cas9 RuvC/HNH and PI domains have also been the targets of modifications. Efforts here focused on directed evolution via yeast and bacterial genetic screening. As can be expected, some of the engineered Cas9 iterations, independent of whichever domain was engineered, also have their drawbacks, mostly in the form of decreased on-target activities [124,125,126,127,128,129,130,131]. However, Cas9 engineering efforts have also led to highly efficient and specific variants like HiFi Cas9 [131]. For an extensive overview of the most prominent Cas9 variants, their respective modified sites, and other features, please refer to Sledzinski et al. [132].

Schmid-Burgk et al. have profiled eight engineered Cas9 as well as spCas9 in terms of activity and specificity via a method termed tagmentation-based tag integration site sequencing (TTISS). Cas9 variants in the profiling included evoCas9, eSpCas9, HiFi Cas9, HypaCas9, Sniper-Cas9, spCas9-HF1 and xCas9. In addition to these variants, they also included their own variant, LZ3 Cas9, obtained by the lentiviral screening of random Cas9 point mutations [131]. In total, 59 sgRNAs in two pools randomly selected from the CRISPR knockout library (GeCKO) were used for the profiling experiments [133]. They were able to show that HiFi Cas9, LZ3 Cas9 and Sniper-Cas9 all had on-target activity similar to spCas9 while improving on-target specificity. However, out of these three newly generated Cas9 iterations, Sniper-Cas9 is the most unspecific. Also worth noting is the fact that their own variant, LZ3 Cas9, exhibits a differential +1 insertion profile when compared to spCas9 [131]. HiFi Cas9, on the other hand, is already used by many groups in the gene editing field, including several groups working on the correction of the mutations underlying EB [134,135,136,137].

Among the most widely used engineered Cas9 enzymes are Cas9 nickases (Cas9n). In contrast to wild-type Cas9, Cas9n only nicks one strand, either the targeted or the untargeted DNA strand. This is achieved by mutating specific amino acids in the endonuclease domains, either HNH or RuvC. The most-used versions are the D10A (targeted strand, HNH) or H840A (untargeted strand, RuvC) Cas9n mutants [67,138,139]. Nicking only one of the DNA strands usually results in traceless repair by the high-fidelity excision base repair (BER) pathway [140]. However, Cas9n mutants, specifically the D10A mutant, are still a valuable tool for gene editing. Cas9n enzymes used in combination with two sgRNAs targeting opposite strands in close proximity can, indeed, create a DSB. This method, termed “double nicking” by Ran et al., can be used for NHEJ- or HDR-based gene editing purposes [141].

Kocher et al. have used plasmid- and RNP-based double-nicking strategies in combination with HDR for the correction of RDEB patient cells with mutations in *COL7A1* [142,143]. Using plasmids encoding Cas9n enzymes, two sgRNA and a double-stranded oligo (dsODN), while also allowing for antibiotic selection, they were able to achieve 89% of HDR-based gene correction efficiencies [142]. The use of a similar but selection-free strategy based on the nucleofection of RNPs as well as single-stranded oligos (ssODN) resulted in 21% and 10% HDR-based correction efficiencies in RDEB patient keratinocytes and fibroblasts. The creation of artificial skin equivalents created with the edited cell populations showed the correct deposition of restored C7 between the dermis and epidermis [143]. In both cases, the use of Cas9n enzymes revealed a superior safety profile when compared to spCas9 [142,143]. Double nicking has also been used by Bischof et al. for the restoration of C17 in JEB keratinocytes [144]. Here, gene reframing via paired nicking using two sgRNAs, flanking a frameshift mutation within exon 52 of the *COL17A1* gene, led to the restoration of C17 in 46% of RNP-treated cells. While the deletion sizes were generally larger than those resulting from single spCas9 treatments, they predominantly consisted of 25- and 37-nucleotide (nt) deletions. In addition, the safety profile of the double-nicking samples was significantly improved. Moreover, 3D skin equivalents created with corrected patient keratinocytes revealed a continuous C17 layer between the epidermis and dermis [144]. Petkovic et al. targeted the same locus with double-nicking RNPs and an ssODN, ultimately achieving up to 18.3% HDR-based gene corrections in JEB patient cells. Including NHEJ-based reframing, the correction efficiency was revealed to be over 40%. Using the same sgRNAs, no off-target cleavage was found via targeted amplicon sequencing of the predicted off-target sites [134].

Prime editing (PE), a relatively new CRISPR-Cas9-based gene editing technique, uses the H840A Cas9 nickase fused to a reverse transcriptase [82]. The sgRNA used for PE, termed prime editing sgRNA (pegRNA), includes a primer binding site (PBS) and a reverse transcriptase template (RTT) on its 3′ end. The RT part of the Cas9n-RT fusion protein uses the RTT to synthesize a new DNA strand at the nicking site on the untargeted strand. The new DNA strand is now incorporated, allowing for the installation of any desired edit at the target locus. The frequency of installing the edit on both DNA strands can be increased by using an additional sgRNA that nicks the initially unedited strand (PE3 system). Once the nick is created, the cellular repair machinery uses the already edited strand as a template for the repair. In their original publication, Anzalone et al. were able to show that PE is relatively free of any of the bystander effects associated with many other gene editing techniques [82].

Hong et al. used PE for the correction of RDEB-causing mutations. Two different heterozygous fibroblast cell lines, Pat1 and Pat2, were transfected with the PE3 system. According to sequencing results, the average correction efficiencies were 10.5% for Pat1 and 5.2% for Pat2. The average correction efficiency for RDEB keratinocytes of a third patient were around 6%. More importantly, C7 was correctly deposited upon grafting of the edited patient fibroblasts on mice [145]. Although the PE technology is highly promising, the correction efficiency in primary cells is generally relatively low. Unfortunately, no commercial Cas9n-RT fusion protein is available to date. In addition, the cell’s repair machinery favours the incorporation of the old, non-edit containing 5′ flap instead of the edit-containing 3’ flap synthesised during the PE process. Having said that, PE is constantly being improved upon, yielding many new PE variants that increase the overall correction efficiencies [146,147,148,149].

Based on a similar idea, Yarnall et al. used the H840A Cas9 nickase for a new method termed programmable addition via site-specific targeting elements (PASTE) [150]. In its first step, PASTE aims to integrate an attB serine integrase attachment site at a desired locus via a PE step using an attachment-site-containing sgRNA (atgRNA) and a Cas9n-RT fusion protein. In a second step, a serine integrase, fused together with the Cas9n and the RT, integrates a transgene into this attachment site. Via this method, they were able to integrate sequences of up to ~36 kilobases (kb) at the target site [150]. Theoretically, this method could be used to integrate whole cDNAs of genes into the genome, either together with an exogenous promoter, or under the control of the respective endogenous promoter. This could be a very promising therapeutic strategy for EB patients, as one molecule would be sufficient for the correction of all mutations in a given gene. However, at this point, PASTE is still in a very early phase and based on the transfection of plasmids carrying the needed molecules. Translation of this method, both in terms of efficacy as well as correction efficiency, to primary EB patient keratinocytes/fibroblasts might be challenging.

Another Cas9 variant extensively used for gene editing purposes is the dead Cas9 (dCas9). In this variant, both nuclease domains are deactivated, resulting in a Cas9 protein that binds to the target DNA but does not create DSBs or nicks [151]. Several dCas9 tools have been developed over time. Initially, dCas9 was used to interfere in gene expression, by either silencing (CRISPRi) or activating (CRISPRa) genes by fusing dCas9 to gene transcriptional repressors or activators, respectively. While gene silencing works quite well in prokaryotes, translation to eukaryotic cells proved to be challenging [151,152,153]. Gene activation via dCas9, on the other hand, has seen many iterations, including systems that can be activated by various ligands [154,155,156,157,158,159,160]. Additionally, dCas9 can also be used for epigenome engineering or chromatin immunoprecipitation [161,162,163,164,165].

However, dCas9 can also be used for gene editing, once again by creating a fusion protein with other enzymes. An interesting approach is the fusion of dCas9 to the FokI endonuclease domain [166,167]. While monomeric FokI is inactive, dimerisation of two units allows for the cleavage of double-stranded DNA. The dCas9-FokI system is therefore used in a manner similar to the Cas9n double-nicking system, as two sgRNAs are needed to target different DNA strands in close proximity. Once binding has occurred, the FokI monomers fused to dCas9 undergo dimerisation. This restores their nuclease activity, leading to the creation of a DSB. Once a DSB is created, it can be utilised for different purposes, including NHEJ-based gene reframing or knockout, as well as HDR-based, traceless repair using a template. To our knowledge, dCas9-FokI has not been used for gene editing in EB patient cells. Nonetheless, it could certainly provide an interesting alternative approach to conventional gene editing with spCas9 or even to double-nicking via Cas9n, especially as the specificity was found to be around 140-fold higher than the former, and around ~4-fold higher than the latter at the loci investigated in the original publications [166,167].

Both dCas9 and Cas9n are extensively used for base editing (BE) [81]. In this system, dCas9 is fused to an ssDNA-nucleobase-modifying enzyme. This enzyme can either be cytidine deaminase (CBE), facilitating a C·G to T·A transition, or adenosine deaminase (ABE), facilitating an A·T to G·C transition. During these transitions, mutagenic intermediates are generated. These intermediates are repaired via cellular repair mechanisms or the replication machinery. Similar to PE, the edit is initially installed only on the untargeted strand. Using a D10A Cas9n instead of a dCas9 increases the changes of installing the edit. Nicking of the target strand recruits the cellular repair machinery. Using the base-edited strand as a template, the edit is then installed on both strands. Unfortunately, base editing is restricted to a certain base-editing window, usually measured by the distance from the PAM. This might make it difficult to target certain mutations. On the other hand, the base-editing mechanism may change all the eligible bases inside this window, possibly creating unwanted bystander nucleotides [81,168,169]. Over the years, many new iterations of the BE system have been developed. For an extensive review of BE, please refer to Liu et al. [170].

BE was used by Osborn et al. for the treatment of two different RDEB patient cell lines, one homozygous and one heterozygous for distinct mutations [171]. In both cases, they aimed for the correction of the underlying mutations in patient fibroblasts via ABEmax, an enhanced adenine base editor. Correction efficiencies on the genomic level were 23.8 and 8.2%, respectively. However, in the heterozygous cell line, two bystander nucleotides within the editing window, as well as modifications of the untargeted allele, were found. 3D skin equivalents created with the homozygous, base-edited patient fibroblasts revealed continuous deposition of C7 between the dermis and epidermis [171]. In addition to PE, Hong et al. also used BE for the correction of *COL7A1* mutations causing RDEB [145]. Using the ABEmax system, they achieved average editing efficiencies of 24.6–30.6% on the DNA level in two different heterozygous fibroblast RDEB cell lines and around 3% in a keratinocyte cell line from a third patient. Indel formation was generally very low, ranging from 0.1–3.3%. Furthermore, intradermal injection of BE-treated patient fibroblasts in immunodeficient mice revealed the correct deposition of C7 between the epidermis and dermis [145]. Naso et al. used mRNA synthesised from a plasmid encoding a 3rd generation cytosine base editor as well as a synthetic sgRNA specifically engineered for an optimised targeting window to edit RDEB patient-induced pluripotent stem cells (iPSC) and fibroblasts [172]. According to next-generation sequencing (NGS), around 59 and 45% of alleles were successfully edited, respectively. Concerning unwanted edits, 19.4 and 4.8% of NGS reads showed the presence of bystander edits in iPSCs and fibroblasts after treatment. However, very little off-target editing was detected at the predicted off-target sites. The group was able to show the correct deposition of C7 at the dermal–epidermal junction in mouse xenografts. Although the signal remained patchy, C7 levels were greatly improved when compared to grafts engineered with untreated patient fibroblasts. Furthermore, transmission electron microscopy (TEM) revealed the presence of anchoring fibrils (AF) at around 50% of the levels detected in WT control grafts [172]. Brooks et al. targeted patient fibroblasts harbouring a mutation in *COL7A1* with the novel ABE8e adenosine base editor system. Similar to Naso et al., base editor mRNA and synthetic sgRNA were used to transfect patient fibroblasts, achieving high levels of successful correction [173].

BE is clearly a powerful gene editing tool, as evidenced by the fact that it has entered the clinical trial phase [174]. Unfortunately, no trials for EB are currently on the way, although the general suitability as well as the relative safety of BE for gene editing in EB patient cells has been demonstrated by the groups mentioned above [145,171,172]. However, two main issues are still worth noting. First, initial BE correction efficiencies were still lacking when compared to more classical CRISPR/Cas9-based gene editing approaches like gene reframing or gene knockout. Having said that, the newest iterations of this technique are definitely more powerful. Second, unwanted bystander edits present in the editing window are of concern, even in the newest BE iterations [170].

### 3.3. Detection of Gene Editing-Related Off-Target Events

Modern gene editing tools allow for the efficient correction of many disease-causing mutations. CRISPR/Cas9 is currently one of the most-used gene editing tools. Many improvements have been made to Cas9(n) and sgRNA specificity over the years, yielding more specific and reliable variants. Naturally, as is the case with any technique that accesses and alters genomic DNA, it is of utmost importance to exclude off-target gene editing. This is especially important because, as discussed above, CRISPR/Cas9 is already used in clinical trials. Reliable and comprehensive off-target prediction/detection is a challenging but recognised aspect of the gene editing field.

A viable but biased strategy to avoid off-target editing is via predictive algorithms. Many bioinformatic tools allow for the prediction of off-targets for a given sgRNA/locus. These predictions are mostly based on sequence similarity and do not always correlate with actual off-target editing. The algorithms these tools are using, however, vary according to the data set(s) that has/have been incorporated. In general, it is advisable to use tools built on several data sets, to obtain a more comprehensive picture [175]. Prominent tools for off-target prediction include CRISPOR, CHOPCHOP, CAS-OFFinder, COSMID, CCTOP, E-CRISP and CRISPRscan [176,177,178,179,180,181,182]. Some companies providing sgRNA synthesis also provide in-built off-target predictions. Off-target predictions can assist in choosing the right technique, approach, or locus for gene editing experiments. Naturally though, off-target sites predicted via in silico, algorithm-based tools require verification after treatment of the respective specimen. A relatively simple way for achieving this is via T7E1 or SURVEYOR assay. These two assays rely on PCR amplification of a region of interest and subsequent denaturation and hybridisation of the products. If indels, created by unwanted CRISPR/Cas9 activity, were present at the (off-target) site, heteroduplexes are formed. These mismatches are then digested via nucleases, creating specific patterns after visualisation of the products. Unfortunately, sensitivity of these assays is relatively low [183,184]. Depending on the hetero- or homozygosity of the locus, the detection of indels is also possible via simple Sanger or NGS. If more details about the nature of off-target cleavage are desired—for example, information about specific indels created—sequencing data can be combined with the tracking of indels by decomposition (TIDE), deconvolution of complex DNA repair (DECODR) or CRISP-ID tool [185,186,187]. More comprehensive and sensitive analyses include targeted amplicon sequencing via NGS, whole-exome sequencing (WES), or even whole-genome sequencing (WGS). However, WES and especially WGS are very cost- and labour-intensive, effectively excluding them as off-target detection tools when aiming for quick confirmation of in silico predictions. Naturally, both WES and WGS provide much more information than amplicon sequencing, including data about potential non-predicted off-target sites, essentially making them unbiased in nature [188]. Many research groups therefore aim for validation of off-target predictions via targeted amplicon sequencing, since it provides a good balance between details gathered and labour/money spent [143,144,189,190,191,192]. Results are usually analysed by a bioinformatician and/or bioinformatic tools such as CRISPResso2 and Cas-Analyzer [193,194].

Prediction and subsequent validation of off-targets is, as mentioned above, a biased approach for off-target detection. Next to these, several methods use a different approach, relying on the detection of DSBs or translocations created by CRISPR/Cas9 in vitro when incubated with genomic DNA in a tube or after treatment of miscellaneous cells. In recent years, as (clinical) gene editing has become more and more relevant, a multitude of these more unbiased sequencing methods has been developed and refined. A summary of selected sequencing detection methods for Cas9/sgRNA-induced DSBs and translocations are listed in Table 3.

With many of the advantages and disadvantages of CRISPR/Cas9 and its derivatives mostly uncovered by now, no significant gene editing experiment aiming at the correction of disease-underlying mutations is complete without some sort of off-target analysis. Depending on the types of experiments as well as their relevance for clinical trials, it might be more than sufficient to predict in silico and validate off-target sites after treatment of the respective cells. Although unbiased approaches are certainly more comprehensive and presumably yield a higher amount of relevant information, they also have their disadvantages. These include, but are not limited to, high false-positive rates and dependence on chromatin state as well as high time and money requirements. It is also worth noting that many of the results yielded by some of the unbiased off-target detection methods will also require locus-specific verification via NGS.

## 4. Conclusions

EB is a rare, monogenetic skin disease with devastating consequences for patients suffering from it’s severe subtypes. Various mutations, in at least 16 different proteins important for skin integrity, lead to a wide variety of symptoms, ranging from smaller wounds at body parts with high friction, to widespread, constantly inflamed wounds leading to the development of SCC. Besides the fact that EB is a rare disease, the variety of EB-causing mutations in all the potentially affected proteins makes gene therapies for EB challenging. However, several attempts have been successfully carried out in the past, including gene replacement therapies for laminin-332 and C7. Excitingly, the first EB-targeting gene replacement pharmaceutical was recently approved by the FDA. Furthermore, in terms of CRISPR/Cas9-based gene editing therapies for EB, many are currently being evaluated in, or are on the way to being evaluated in clinical trials, including BE-based therapies.

In terms of future gene editing approaches, the CRISPR/Cas9 toolbox has been greatly expanded since its discovery. Engineering of the Cas9 protein and sgRNAs have yielded more efficient and safer variants. Additionally, many research groups have developed modifications of the CRISPR/Cas9 system, including BE, PE, and PASTE. In parallel, validation of the safety aspects has been made easier and more efficient over time, with several new unbiased off-target techniques and advanced prediction algorithms available for use.

Even though EB is a rare disease with only around 500,000 people affected worldwide, many interesting and sophisticated gene therapies have been developed over the course of the last decade. Similar to gene therapies for other diseases, therapies for EB will probably adopt the more specific Cas9 variants and methods recently developed. Ultimately, this development will lead to more approved therapies, similar to Vyjuvek™. In conclusion, gene therapies, and especially gene editing, have proven to be the most promising and safest approaches for causal correction of EB-related mutations and monogenetic diseases in general. In case of CRISPR/Cas9, several remarkable engineering developments have brought this tool to the forefront of future gene editing efforts.

## Figures and Tables

**Table 1:** Completed, ongoing, and terminated gene therapy clinical trials for epidermolysis bullosa (www.clinicaltrials.gov; accessed on 15 November 2023).

EB Type	Gene	Strategy	Phase	Status	Sponsor	NCT ID
JEB	*LAMB3*	Transplantation of autologous gene-corrected epidermal sheets	Phase I/II	Completed	Department of Biomedical Sciences, University of Modena and Reggio Emilia, Modena, Italy	Not listed [39]
JEB	*LAMB3*	Transplantation of autologous gene-corrected epidermal sheets	Phase I/II	Completed	Department of Biomedical Sciences, University of Modena and Reggio Emilia, Modena, Italy	Not listed [40]
JEB	*LAMB3*	Transplantation of autologous gene-corrected epidermal sheets	Phase I/II	Completed	University Hospital of the Paracelsus Medical University, Salzburg, Salzburg, Austria	Case study, not listed [41]
JEB	*LAMB3*	Transplantation of autologous gene-corrected epidermal sheets	Phase II/III	Recruiting	Holostem Terapie Avanzate s.r.l., Modena, Italy	NCT05111600
JEB	*COL17A1*	Transplantation of autologous gene-corrected epidermal sheets	Phase I/II	Terminated (no patient ongoing/completed)	Holostem Terapie Avanzate s.r.l., Modena, Italy	NCT03490331
RDEB	*COL7A1*	Transplantation of autologous gene-corrected epidermal sheets	Phase I/II	Completed	Abeona Therapeutics, Inc., Cleveland, OH, USA	NCT01263379
RDEB	*COL7A1*	Transplantation of autologous gene-corrected epidermal sheets	Phase III	Completed	Abeona Therapeutics, Inc., Cleveland, OH, USA	NCT04227106
RDEB	*COL7A1*	Transplantation of autologous gene-corrected epidermal sheets	Phase III	Recruiting	Abeona Therapeutics, Inc., Cleveland, OH, USA	NCT05725018
RDEB	*COL7A1*	Intradermal injection of autologous gene-corrected fibroblasts	Phase I	Completed	King’s College, London, UK	NCT02493816
RDEB	*COL7A1*	Intradermal injection of autologous gene-corrected fibroblasts	Phase I/II	Terminated (subjects transferred to NCT04213261)	Castle Creek Biosciences, LLC., Exton, PA, USA	NCT02810951
RDEB	*COL7A1*	Intradermal injection of autologous gene-corrected fibroblasts	Phase III	Active, not recruiting	Castle Creek Biosciences, LLC., Exton, PA, USA	NCT04213261
RDEB	*COL7A1*	Replication-incompetent HSV-1 carrying *COL7A1*, topical application (in vivo)	Phase I/II	Completed	Krystal Biotech, Inc., Pittsburgh, PA, USA	NCT03536143
RDEB (DDEB)	*COL7A1*	Replication-incompetent HSV-1 carrying *COL7A1*, topical application (in vivo)	Phase III	Completed	Krystal Biotech, Inc., Pittsburgh, PA, USA	NCT04491604
RDEB (DDEB)	*COL7A1*	Replication-incompetent HSV-1 carrying *COL7A1*, topical application (in vivo)	Long-time follow-up study	Recruiting	Krystal Biotech, Inc., Pittsburgh, PA, USA	NCT04917887
RDEB	*COL7A1*	Transplantation of autologous gene-corrected keratinocyte sheets	Phase I/II	Active, not recruiting	Institut National de la Santé Et de la Recherche Médicale, Paris, France	NCT04186650
RDEB	*COL7A1*	Transplantation of autologous gene-corrected epidermal sheets	Phase I/II	Terminated (replaced by new study in progress)	Holostem Terapie Avanzate s.r.l., Modena, Italy	NCT02984085
RDEB DDEB	*COL7A1*	ASO QR-313, topical application (in vivo)	Phase l/II	Terminated (low enrolment)	Phoenicis Therapeutics, Hingham, MA, USA	NCT03605069

**Table 2:** Impact of chemical modifications, extensions/truncations, and substitutions on sgRNA specificity.

Mod. Type	Specific Modification	Mod. Position	Cell Type	Targeted Loci	Type of Molecule	Effect on Efficiency	Ref.
**Chemical modification**	2’-O-methyl/2’O-methyl 3’phosphothiorate/2’-O-methyl-3’-thioPACE	5’ and 3’ ends of sgRNA	K562 cells/primary T cells/hematopoietic stem cells	CCR5/HBB/IL2RG	sgRNA + Cas9 plasmid/mRNA	Increased	Hendel et al. [102]
	Diverse chemical modifications	Whole crRNA	HEK293 cells	GFP (integrated)	sgRNA + Cas9 protein	Increased	O’Reilly et al. [103]
		spacer seed region				Decreased	
	2’O-methyl	5’ and 3’ ends of crRNA	HEK293 cells	VEGF-A	sgRNA + Cas9 mRNA/protein	Increased	Rahdar et al. [98]
	2’-fluoro/S-constrained ethyl	PAM-distal spacer region/tracrRNA-binding spacer region					
	2’-fluoro/S-constrained ethyl	spacer seed region				Decreased	
	Phosphorothioate	Whole crRNA				Increased	
	2’O-methyl 3’phosphothiorate	5’ and 3’ ends of crRNA, tracrRNA or sgRNA	K562 cells	PPIB/PSMD7/PSMD11	sgRNA + Cas9 mRNA/protein	Increased	Basila et al. [104]
	2’O-methyl 3’phosphothiorate	PAM-distal spacer region/tracrRNA-binding spacer region	HEK293 cells/mouse liver cells (in vivo)	EMX1/GFP (integrated)/HBB/PCSK9	sgRNA + Cas9 mRNA/protein	Increased	Yin et al. [105]
		spacer seed region					
	2’-fluoro/phosphorothioate	PAM-distal spacer region/tracrRNA-binding spacer region				Decreased	
		spacer seed region					
	2’O-methyl 3’phosphothiorate	5’ and 3’ ends of sgRNA/PAM-distal spacer region/tracrRNA-binding spacer region	Mouse and rat liver cells (in vivo)	Ttr	sgRNA + Cas9 mRNA/protein	Increased	Finn et al. [106]
	2’O-methyl 3’phosphothiorate	PAM-distal spacer region/tracrRNA-binding spacer region	HEK293 cells/Human embryonic stem cells	EMX1/HBB/VEGFA	sgRNA + Cas9 protein	Increased	Mir et al. [107]
		spacer seed region				Decreased	
	2’-fluoro/Phosphorothioate	PAM-distal spacer region/tracrRNA-binding spacer region				Increased	
		spacer seed region				Decreased	
	2’-O-methyl-3’-thioPACE	Central region of crRNA	K562 cells	CLTA4/HBB/IL2RG/VEGFA	sgRNA+ Cas9 protein	Increased	Ryan et al. [108]
**Truncation/** **Extension**	Truncated sgRNAs (tru-gRNA)	5’ end of sgRNA	HEK293 cells/UO2S cell	EMX1/GFP (integrated)/VEGFA	Plasmids	Increased	Fu et al. [109]
	Extension of sgRNA (5’-GGX20)	5’ end of sgRNA	K562 cells/HeLa cells	EMX1/VEGF-A	Plasmids	Increased	Cho et al. [110]
	Hairpin extension (hp-sgRNA)	5’ end of sgRNA	HEK293 cells	EMX1/VEGFA	Plasmids	Increased	Kocak et al. [111]
**Substitution**	Bridged/locked nucleic acid substitutions (BNA/LNA)	Central region crRNA	HeLa cells/U2OS cells	EMX1/WAS	sgRNA + Cas9 protein	Increased	Cromwell et al. [112]
	DNA substitution	5’ end of sgRNA	HEK293 cells	AAVS1	sgRNA/sgDNA + Cas9 protein	Increased	Rueda et al. [113]
	DNA substitution	5’ end of sgRNA	HEK293 cells/U2OS cells	EMX1/GFP (integrated)/VEGFAA	sgRNA/sgDNA + Cas9 protein	Increased	Yin et al. [114]
	DNA substitution	5’ end of sgRNA/tracrRNA-binding spacer region	In vitro	GFP (Plasmid)	sg/sgDNA + Cas9 protein	Increased	Kartje et al. [115]

**Table 3:** Summary of selected Cas9-induced off-target detection methods.

Method	Acronym	Description	Detection of	Setting	Advantages	Disadvantages	Ref.
**ChIP-seq**	Chromatin Immunoprecipitation sequencing	Mapping of dCas9/sgRNA binding sites	Cas9 binding	Cells	Unbiased	Binding of dCas9/sgRNA does not necessarily correlate with cleavage by Cas9/sgRNA	Kuscu et al. [195]
**HTGTS**	High-throughput genome-wide translocation sequencing	Translocation detection via bait-prey primers	Repair products	Cells	High sensitivity	Limited by chromatin accessibility	Frock et al. [196]
**Digenome-seq**	In vitro nuclease-digested genome sequencing	Digestion of purified DNA with Cas9/sgRNA	DSBs	DNA	Unbiased/High sensitivity	False positives	Kim et al. [197]
**GUIDE-seq**	Genome-wide, unbiased identification of DSBs enabled by sequencing	Integration of oligonucleotides at DSBs created by Cas9/sgRNA	Repair products	DNA	High sensitivity/Low false positive rate	False negatives	Tsai et al. [198]
**SITE-seq**	Selective enrichment and identification of tagged genomic DNA ends by sequencing	Enrichment and digestion of purified DNA with Cas9/sgRNA	DSBs	DNA	No reference genome required	Low sensitivity/False positives	Cameron et al. [199]
**CIRCLE-seq**	Circularisation for in vitro reporting of cleavage effects by sequencing	Incubation of circularised and sheared genomic DNA with Cas9/sgRNA	DSBs	DNA	High sensitivity	False positives	Tsai et al. [200]
**BLISS**	Breaks labelling in situ and sequencing	Integration of oligonucleotides at DSBs created by Cas9/sgRNA	DSBs	Cells or tissue	Low input needed		Yan et al. [201]
**DIG-seq**	(Digenome derivative)	Enrichment and digestion of purified DNA with Cas9/sgRNA	DSBs	DNA	Higher validation rate than Digenome-seq		Kim et al. [202]
**TTISS**	Tagmentation-based tag integration site sequencing	In situ labelling of DSBs created by Cas/sgRNA	DSBs	Cells or tissue	Multiplexing possible		Schmid-Burgk et al. [131]
**DISCOVER-seq**	Discovery of in situ Cas off-targets and verification by sequencing	Enrichment and digestion of purified DNA with Cas9/sgRNA	DSBs	Cells or tissue	High sensitivity	False positives	Wienert et al. [203]
**CAST-seq**	Chromosomal aberrations analysis by single targeted linker-mediated PCR sequencing	Translocation detection via bait-prey primers	Repair product	Cells	Unbiased/Quantitative method		Turchiano et al. [204]

## References

[1] Has C., Bauer J.W., Bodemer C., Bolling M.C., Bruckner-Tuderman L., Diem A., Fine J.-D., Heagerty A., Hovnanian A., Marinkovich M.P. (2020). Consensus reclassification of inherited epidermolysis bullosa and other disorders with skin fragility. Br. J. Dermatol..

[2] Tang J.Y., Marinkovich M.P., Lucas E., Gorell E., Chiou A., Lu Y., Gillon J., Patel D., Rudin D. (2021). A systematic literature review of the disease burden in patients with recessive dystrophic epidermolysis bullosa. Orphanet J. Rare Dis..

[3] Baardman R., Bolling M.C. (2022). The importance of accurate epidemiological data of epidermolysis bullosa. Br. J. Dermatol..

[4] Petrof G., Papanikolaou M., Martinez A.E., Mellerio J.E., McGrath J.A., Bardhan A., Harper N., Heagerty A., Ogboli M., Chiswell C. (2022). The epidemiology of epidermolysis bullosa in England and Wales: Data from the national epidermolysis bullosa database. Br. J. Dermatol..

[5] Baardman R., Yenamandra V.K., Duipmans J.C., Pasmooij A.M.G., Jonkman M.F., van den Akker P.C., Bolling M.C. (2021). Novel insights into the epidemiology of epidermolysis bullosa (EB) from the Dutch EB Registry: EB more common than previously assumed?. J. Eur. Acad. Dermatol. Venereol..

[6] Sprecher E. (2010). Epidermolysis bullosa simplex. Dermatol. Clin..

[7] Condrat I., He Y., Cosgarea R., Has C. (2018). Junctional Epidermolysis Bullosa: Allelic Heterogeneity and Mutation Stratification for Precision Medicine. Front. Med..

[8] Pfendner E.G., Lucky A.W., Adam M.P., Feldman J., Mirzaa G.M., Pagon R.A., Wallace S.E., Bean L.J.H., Gripp K.W., Amemiya A. (1993). Junctional Epidermolysis Bullosa. GeneReviews ((R)).

[9] Youssefian L., Vahidnezhad H., Uitto J., Adam M.P., Feldman J., Mirzaa G.M., Pagon R.A., Wallace S.E., Bean L.J.H., Gripp K.W., Amemiya A. (1993). Kindler Syndrome. GeneReviews ((R)).

[10] Kocher T., Petkovic I., Bischof J., Koller U. (2022). Current developments in gene therapy for epidermolysis bullosa. Expert. Opin. Biol. Ther..

[11] Carter D.M., Lin A.N., Varghese M.C., Caldwell D., Pratt L.A., Eisinger M. (1987). Treatment of junctional epidermolysis bullosa with epidermal autografts. J. Am. Acad. Dermatol..

[12] Carter D.M., Lin A.N. (1988). Wound healing and epidermolysis bullosa. Arch. Dermatol..

[13] Jonkman M.F., Scheffer H., Stulp R., Pas H.H., Nijenhuis M., Heeres K., Owaribe K., Pulkkinen L., Uitto J. (1997). Revertant mosaicism in epidermolysis bullosa caused by mitotic gene conversion. Cell.

[14] Pasmooij A.M., Nijenhuis M., Brander R., Jonkman M.F. (2012). Natural gene therapy may occur in all patients with generalized non-Herlitz junctional epidermolysis bullosa with COL17A1 mutations. J. Investig. Dermatol..

[15] van den Akker P.C., Bolling M.C., Pasmooij A.M.G. (2022). Revertant Mosaicism in Genodermatoses: Natural Gene Therapy Right before Your Eyes. Biomedicines.

[16] Gostynski A., Pasmooij A.M., Jonkman M.F. (2014). Successful therapeutic transplantation of revertant skin in epidermolysis bullosa. J. Am. Acad. Dermatol..

[17] Marinkovich M.P., Tang J.Y. (2019). Gene Therapy for Epidermolysis Bullosa. J. Investig. Dermatol..

[18] Falabella A.F., Valencia I.C., Eaglstein W.H., Schachner L.A. (2000). Tissue-engineered skin (Apligraf) in the healing of patients with epidermolysis bullosa wounds. Arch. Dermatol..

[19] Fivenson D.P., Scherschun L., Choucair M., Kukuruga D., Young J., Shwayder T. (2003). Graftskin therapy in epidermolysis bullosa. J. Am. Acad. Dermatol..

[20] Wilkins L.M., Watson S.R., Prosky S.J., Meunier S.F., Parenteau N.L. (1994). Development of a bilayered living skin construct for clinical applications. Biotechnol. Bioeng..

[21] Petrof G., Martinez-Queipo M., Mellerio J.E., Kemp P., McGrath J.A. (2013). Fibroblast cell therapy enhances initial healing in recessive dystrophic epidermolysis bullosa wounds: Results of a randomized, vehicle-controlled trial. Br. J. Dermatol..

[22] Venugopal S.S., Yan W., Frew J.W., Cohn H.I., Rhodes L.M., Tran K., Melbourne W., Nelson J.A., Sturm M., Fogarty J. (2013). A phase II randomized vehicle-controlled trial of intradermal allogeneic fibroblasts for recessive dystrophic epidermolysis bullosa. J. Am. Acad. Dermatol..

[23] Conget P., Rodriguez F., Kramer S., Allers C., Simon V., Palisson F., Gonzalez S., Yubero M.J. (2010). Replenishment of type VII collagen and re-epithelialization of chronically ulcerated skin after intradermal administration of allogeneic mesenchymal stromal cells in two patients with recessive dystrophic epidermolysis bullosa. Cytotherapy.

[24] Petrof G., Lwin S.M., Martinez-Queipo M., Abdul-Wahab A., Tso S., Mellerio J.E., Slaper-Cortenbach I., Boelens J.J., Tolar J., Veys P. (2015). Potential of Systemic Allogeneic Mesenchymal Stromal Cell Therapy for Children with Recessive Dystrophic Epidermolysis Bullosa. J. Investig. Dermatol..

[25] El-Darouti M., Fawzy M., Amin I., Abdel Hay R., Hegazy R., Gabr H., El Maadawi Z. (2016). Treatment of dystrophic epidermolysis bullosa with bone marrow non-hematopoeitic stem cells: A randomized controlled trial. Dermatol. Ther..

[26] Wagner J.E., Ishida-Yamamoto A., McGrath J.A., Hordinsky M., Keene D.R., Woodley D.T., Chen M., Riddle M.J., Osborn M.J., Lund T. (2010). Bone marrow transplantation for recessive dystrophic epidermolysis bullosa. N. Engl. J. Med..

[27] Heinonen S., Mannikko M., Klement J.F., Whitaker-Menezes D., Murphy G.F., Uitto J. (1999). Targeted inactivation of the type VII collagen gene (Col7a1) in mice results in severe blistering phenotype: A model for recessive dystrophic epidermolysis bullosa. J. Cell Sci..

[28] Tolar J., Ishida-Yamamoto A., Riddle M., McElmurry R.T., Osborn M., Xia L., Lund T., Slattery C., Uitto J., Christiano A.M. (2009). Amelioration of epidermolysis bullosa by transfer of wild-type bone marrow cells. Blood.

[29] Kopp J., Horch R.E., Stachel K.D., Holter W., Kandler M.A., Hertzberg H., Rascher W., Campean V., Carbon R., Schneider H. (2005). Hematopoietic stem cell transplantation and subsequent 80% skin exchange by grafts from the same donor in a patient with Herlitz disease. Transplantation.

[30] Hammersen J., Has C., Naumann-Bartsch N., Stachel D., Kiritsi D., Soder S., Tardieu M., Metzler M., Bruckner-Tuderman L., Schneider H. (2016). Genotype, Clinical Course, and Therapeutic Decision Making in 76 Infants with Severe Generalized Junctional Epidermolysis Bullosa. J. Investig. Dermatol..

[31] Bornert O., Peking P., Bremer J., Koller U., van den Akker P.C., Aartsma-Rus A., Pasmooij A.M., Murauer E.M., Nystrom A. (2017). RNA-based therapies for genodermatoses. Exp. Dermatol..

[32] Hainzl S., Trattner L., Liemberger B., Bischof J., Kocher T., Ablinger M., Nystrom A., Obermayer A., Klausegger A., Guttmann-Gruber C. (2024). Splicing Modulation via Antisense Oligonucleotides in Recessive Dystrophic Epidermolysis Bullosa. Int. J. Mol. Sci..

[33] Bremer J., Bornert O., Nystrom A., Gostynski A., Jonkman M.F., Aartsma-Rus A., van den Akker P.C., Pasmooij A.M. (2016). Antisense Oligonucleotide-mediated Exon Skipping as a Systemic Therapeutic Approach for Recessive Dystrophic Epidermolysis Bullosa. Mol. Ther. Nucleic Acids.

[34] Bornert O., Hogervorst M., Nauroy P., Bischof J., Swildens J., Athanasiou I., Tufa S.F., Keene D.R., Kiritsi D., Hainzl S. (2021). QR-313, an Antisense Oligonucleotide, Shows Therapeutic Efficacy for Treatment of Dominant and Recessive Dystrophic Epidermolysis Bullosa: A Preclinical Study. J. Investig. Dermatol..

[35] Turczynski S., Titeux M., Tonasso L., Decha A., Ishida-Yamamoto A., Hovnanian A. (2016). Targeted Exon Skipping Restores Type VII Collagen Expression and Anchoring Fibril Formation in an In Vivo RDEB Model. J. Investig. Dermatol..

[36] Ablinger M., Lettner T., Friedl N., Potocki H., Palmetzhofer T., Koller U., Illmer J., Liemberger B., Hainzl S., Klausegger A. (2021). Personalized Development of Antisense Oligonucleotides for Exon Skipping Restores Type XVII Collagen Expression in Junctional Epidermolysis Bullosa. Int. J. Mol. Sci..

[37] Humbert O., Davis L., Maizels N. (2012). Targeted gene therapies: Tools, applications, optimization. Crit. Rev. Biochem. Mol. Biol..

[38] Koller U. (2023). Gene therapy advances shine the spotlight on epidermolysis bullosa, bringing hope to patients. Mol. Ther..

[39] Mavilio F., Pellegrini G., Ferrari S., Di Nunzio F., Di Iorio E., Recchia A., Maruggi G., Ferrari G., Provasi E., Bonini C. (2006). Correction of junctional epidermolysis bullosa by transplantation of genetically modified epidermal stem cells. Nat. Med..

[40] Hirsch T., Rothoeft T., Teig N., Bauer J.W., Pellegrini G., De Rosa L., Scaglione D., Reichelt J., Klausegger A., Kneisz D. (2017). Regeneration of the entire human epidermis using transgenic stem cells. Nature.

[41] Bauer J.W., Koller J., Murauer E.M., De Rosa L., Enzo E., Carulli S., Bondanza S., Recchia A., Muss W., Diem A. (2017). Closure of a Large Chronic Wound through Transplantation of Gene-Corrected Epidermal Stem Cells. J. Investig. Dermatol..

[42] De Rosa L., Carulli S., Cocchiarella F., Quaglino D., Enzo E., Franchini E., Giannetti A., De Santis G., Recchia A., Pellegrini G. (2014). Long-term stability and safety of transgenic cultured epidermal stem cells in gene therapy of junctional epidermolysis bullosa. Stem Cell Rep..

[43] De Rosa L., Enzo E., Zardi G., Bodemer C., Magnoni C., Schneider H., De Luca M. (2021). Hologene 5: A Phase II/III Clinical Trial of Combined Cell and Gene Therapy of Junctional Epidermolysis Bullosa. Front. Genet..

[44] De Rosa L., De Luca M. (2022). The joint battle to tackle epidermolysis bullosa through gene therapy. Trends Mol. Med..

[45] Barrandon Y., Green H. (1987). Three clonal types of keratinocyte with different capacities for multiplication. Proc. Natl. Acad. Sci. USA.

[46] Kueckelhaus M., Rothoeft T., De Rosa L., Yeni B., Ohmann T., Maier C., Eitner L., Metze D., Losi L., Secone Seconetti A. (2021). Transgenic Epidermal Cultures for Junctional Epidermolysis Bullosa—5-Year Outcomes. N. Engl. J. Med..

[47] Siprashvili Z., Nguyen N.T., Gorell E.S., Loutit K., Khuu P., Furukawa L.K., Lorenz H.P., Leung T.H., Keene D.R., Rieger K.E. (2016). Safety and Wound Outcomes Following Genetically Corrected Autologous Epidermal Grafts in Patients with Recessive Dystrophic Epidermolysis Bullosa. JAMA.

[48] Eichstadt S., Barriga M., Ponakala A., Teng C., Nguyen N.T., Siprashvili Z., Nazaroff J., Gorell E.S., Chiou A.S., Taylor L. (2019). Phase 1/2a clinical trial of gene-corrected autologous cell therapy for recessive dystrophic epidermolysis bullosa. JCI Insight.

[49] So J.Y., Nazaroff J., Iwummadu C.V., Harris N., Gorell E.S., Fulchand S., Bailey I., McCarthy D., Siprashvili Z., Marinkovich M.P. (2022). Long-term safety and efficacy of gene-corrected autologous keratinocyte grafts for recessive dystrophic epidermolysis bullosa. Orphanet J. Rare Dis..

[50] Benati D., Miselli F., Cocchiarella F., Patrizi C., Carretero M., Baldassarri S., Ammendola V., Has C., Colloca S., Del Rio M. (2018). CRISPR/Cas9-Mediated In Situ Correction of LAMB3 Gene in Keratinocytes Derived from a Junctional Epidermolysis Bullosa Patient. Mol. Ther..

[51] De Rosa L., Secone Seconetti A., De Santis G., Pellacani G., Hirsch T., Rothoeft T., Teig N., Pellegrini G., Bauer J.W., De Luca M. (2019). Laminin 332-Dependent YAP Dysregulation Depletes Epidermal Stem Cells in Junctional Epidermolysis Bullosa. Cell Rep..

[52] Jackow J., Titeux M., Portier S., Charbonnier S., Ganier C., Gaucher S., Hovnanian A. (2016). Gene-Corrected Fibroblast Therapy for Recessive Dystrophic Epidermolysis Bullosa using a Self-Inactivating COL7A1 Retroviral Vector. J. Investig. Dermatol..

[53] Lwin S.M., Syed F., Di W.L., Kadiyirire T., Liu L., Guy A., Petrova A., Abdul-Wahab A., Reid F., Phillips R. (2019). Safety and early efficacy outcomes for lentiviral fibroblast gene therapy in recessive dystrophic epidermolysis bullosa. JCI Insight.

[54] Marinkovich M., Lane A., Sridhar K., Keene D., Malyala A., Maslowski J. (2018). 591 A phase 1/2 study of genetically-corrected, collagen VII expressing autologous human dermal fibroblasts injected into the skin of patients with recessive dystrophic epidermolysis bullosa (RDEB). J. Investig. Dermatol..

[55] Gurevich I., Agarwal P., Zhang P., Dolorito J.A., Oliver S., Liu H., Reitze N., Sarma N., Bagci I.S., Sridhar K. (2022). In vivo topical gene therapy for recessive dystrophic epidermolysis bullosa: A phase 1 and 2 trial. Nat. Med..

[56] Welponer T., Prodinger C., Pinon-Hofbauer J., Hintersteininger A., Breitenbach-Koller H., Bauer J.W., Laimer M. (2021). Clinical Perspectives of Gene-Targeted Therapies for Epidermolysis Bullosa. Dermatol. Ther..

[57] Mullard A. (2023). FDA approves first topical gene therapy. Nat. Rev. Drug Discov..

[58] Guide S.V., Gonzalez M.E., Bagci I.S., Agostini B., Chen H., Feeney G., Steimer M., Kapadia B., Sridhar K., Quesada Sanchez L. (2022). Trial of Beremagene Geperpavec (B-VEC) for Dystrophic Epidermolysis Bullosa. N. Engl. J. Med..

[59] Kotterman M.A., Chalberg T.W., Schaffer D.V. (2015). Viral Vectors for Gene Therapy: Translational and Clinical Outlook. Annu. Rev. Biomed. Eng..

[60] Hacein-Bey-Abina S., Garrigue A., Wang G.P., Soulier J., Lim A., Morillon E., Clappier E., Caccavelli L., Delabesse E., Beldjord K. (2008). Insertional oncogenesis in 4 patients after retrovirus-mediated gene therapy of SCID-X1. J. Clin. Investig..

[61] Biasco L., Baricordi C., Aiuti A. (2012). Retroviral integrations in gene therapy trials. Mol. Ther..

[62] (2021). Gene therapy needs a long-term approach. Nat. Med..

[63] March O.P., Kocher T., Koller U. (2020). Context-Dependent Strategies for Enhanced Genome Editing of Genodermatoses. Cells.

[64] Lieber M.R. (2010). The mechanism of double-strand DNA break repair by the nonhomologous DNA end-joining pathway. Annu. Rev. Biochem..

[65] Pannunzio N.R., Li S., Watanabe G., Lieber M.R. (2014). Non-homologous end joining often uses microhomology: Implications for alternative end joining. DNA Repair.

[66] Rodgers K., McVey M. (2016). Error-Prone Repair of DNA Double-Strand Breaks. J. Cell Physiol..

[67] Jinek M., Chylinski K., Fonfara I., Hauer M., Doudna J.A., Charpentier E. (2012). A programmable dual-RNA-guided DNA endonuclease in adaptive bacterial immunity. Science.

[68] Makarova K.S., Wolf Y.I., Iranzo J., Shmakov S.A., Alkhnbashi O.S., Brouns S.J.J., Charpentier E., Cheng D., Haft D.H., Horvath P. (2020). Evolutionary classification of CRISPR-Cas systems: A burst of class 2 and derived variants. Nat. Rev. Microbiol..

[69] Zetsche B., Gootenberg J.S., Abudayyeh O.O., Slaymaker I.M., Makarova K.S., Essletzbichler P., Volz S.E., Joung J., van der Oost J., Regev A. (2015). Cpf1 is a single RNA-guided endonuclease of a class 2 CRISPR-Cas system. Cell.

[70] Bandyopadhyay A., Kancharla N., Javalkote V.S., Dasgupta S., Brutnell T.P. (2020). CRISPR-Cas12a (Cpf1): A Versatile Tool in the Plant Genome Editing Tool Box for Agricultural Advancement. Front. Plant Sci..

[71] Liang M., Li Z., Wang W., Liu J., Liu L., Zhu G., Karthik L., Wang M., Wang K.F., Wang Z. (2019). A CRISPR-Cas12a-derived biosensing platform for the highly sensitive detection of diverse small molecules. Nat. Commun..

[72] Wang Y.M., Liu K.W.I., Sutrisnoh N.A.B., Srinivasan H., Zhang J.Y., Li J., Zhang F., Lalith C.R.J., Xing H.Y., Shanmugam R. (2018). Systematic evaluation of CRISPR-Cas systems reveals design principles for genome editing in human cells. Genome Biol..

[73] Moreno-Mateos M.A., Fernandez J.P., Rouet R., Vejnar C.E., Lane M.A., Mis E., Khokha M.K., Doudna J.A., Giraldez A.J. (2017). CRISPR-Cpf1 mediates efficient homology-directed repair and temperature-controlled genome editing. Nat. Commun..

[74] Fagerlund R.D., Staals R.H., Fineran P.C. (2015). The Cpf1 CRISPR-Cas protein expands genome-editing tools. Genome Biol..

[75] Modrzejewski D., Hartung F., Lehnert H., Sprink T., Kohl C., Keilwagen J., Wilhelm R. (2020). Which Factors Affect the Occurrence of Off-Target Effects Caused by the Use of CRISPR/Cas: A Systematic Review in Plants. Front. Plant Sci..

[76] Alok A., Sandhya D., Jogam P., Rodrigues V., Bhati K.K., Sharma H., Kumar J. (2020). The Rise of the CRISPR/Cpf1 System for Efficient Genome Editing in Plants. Front. Plant Sci..

[77] Senthilnathan R., Ilangovan I., Kunale M., Easwaran N., Ramamoorthy S., Veeramuthu A., Kodiveri Muthukaliannan G. (2023). An update on CRISPR-Cas12 as a versatile tool in genome editing. Mol. Biol. Rep..

[78] Badon I.W., Oh Y., Kim H.J., Lee S.H. (2024). Recent application of CRISPR-Cas12 and OMEGA system for genome editing. Mol. Ther..

[79] Karvelis T., Druteika G., Bigelyte G., Budre K., Zedaveinyte R., Silanskas A., Kazlauskas D., Venclovas C., Siksnys V. (2021). Transposon-associated TnpB is a programmable RNA-guided DNA endonuclease. Nature.

[80] Saito M., Xu P., Faure G., Maguire S., Kannan S., Altae-Tran H., Vo S., Desimone A., Macrae R.K., Zhang F. (2023). Fanzor is a eukaryotic programmable RNA-guided endonuclease. Nature.

[81] Komor A.C., Kim Y.B., Packer M.S., Zuris J.A., Liu D.R. (2016). Programmable editing of a target base in genomic DNA without double-stranded DNA cleavage. Nature.

[82] Anzalone A.V., Randolph P.B., Davis J.R., Sousa A.A., Koblan L.W., Levy J.M., Chen P.J., Wilson C., Newby G.A., Raguram A. (2019). Search-and-replace genome editing without double-strand breaks or donor DNA. Nature.

[83] Huang K., Zapata D., Tang Y., Teng Y., Li Y. (2022). In vivo delivery of CRISPR-Cas9 genome editing components for therapeutic applications. Biomaterials.

[84] Ferraresi M., Panzieri D.L., Leoni S., Cappellini M.D., Kattamis A., Motta I. (2023). Therapeutic perspective for children and young adults living with thalassemia and sickle cell disease. Eur. J. Pediatr..

[85] Demirci S., Leonard A., Essawi K., Tisdale J.F. (2021). CRISPR-Cas9 to induce fetal hemoglobin for the treatment of sickle cell disease. Mol. Ther. Methods Clin. Dev..

[86] Frangoul H., Altshuler D., Cappellini M.D., Chen Y.S., Domm J., Eustace B.K., Foell J., de la Fuente J., Grupp S., Handgretinger R. (2021). CRISPR-Cas9 Gene Editing for Sickle Cell Disease and beta-Thalassemia. N. Engl. J. Med..

[87] Hunt J.M.T., Samson C.A., Rand A.D., Sheppard H.M. (2023). Unintended CRISPR-Cas9 editing outcomes: A review of the detection and prevalence of structural variants generated by gene-editing in human cells. Hum. Genet..

[88] Pattanayak V., Lin S., Guilinger J.P., Ma E., Doudna J.A., Liu D.R. (2013). High-throughput profiling of off-target DNA cleavage reveals RNA-programmed Cas9 nuclease specificity. Nat. Biotechnol..

[89] Zhang X.H., Tee L.Y., Wang X.G., Huang Q.S., Yang S.H. (2015). Off-target Effects in CRISPR/Cas9-mediated Genome Engineering. Mol. Ther-Nucl. Acids.

[90] Josephs E.A., Kocak D.D., Fitzgibbon C.J., McMenemy J., Gersbach C.A., Marszalek P.E. (2015). Structure and specificity of the RNA-guided endonuclease Cas9 during DNA interrogation, target binding and cleavage. Nucleic Acids Res..

[91] Wang Y., Wang M., Zheng T., Hou Y., Zhang P., Tang T., Wei J., Du Q. (2020). Specificity profiling of CRISPR system reveals greatly enhanced off-target gene editing. Sci. Rep..

[92] Fu R.J., He W., Dou J.Z., Villarreal O.D., Bedford E., Wang H.E., Hou C., Zhang L., Wang Y.L., Ma D.C. (2022). Systematic decomposition of sequence determinants governing CRISPR/Cas9 specificity. Nat. Commun..

[93] O’Geen H., Yu A.S., Segal D.J. (2015). How specific is CRISPR/Cas9 really?. Curr. Opin. Chem. Biol..

[94] Anders C., Niewoehner O., Duerst A., Jinek M. (2014). Structural basis of PAM-dependent target DNA recognition by the Cas9 endonuclease. Nature.

[95] Nishimasu H., Ran F.A., Hsu P.D., Konermann S., Shehata S.I., Dohmae N., Ishitani R., Zhang F., Nureki O. (2014). Crystal Structure of Cas9 in Complex with Guide RNA and Target DNA. Cell.

[96] Sternberg S.H., Redding S., Jinek M., Greene E.C., Doudna J.A. (2014). DNA interrogation by the CRISPR RNA-guided endonuclease Cas9. Nature.

[97] Konermann S., Brigham M.D., Trevino A.E., Joung J., Abudayyeh O.O., Barcena C., Hsu P.D., Habib N., Gootenberg J.S., Nishimasu H. (2015). Genome-scale transcriptional activation by an engineered CRISPR-Cas9 complex. Nature.

[98] Rahdar M., McMahon M.A., Prakash T.P., Swayze E.E., Bennett C.F., Cleveland D.W. (2015). Synthetic CRISPR RNA-Cas9-guided genome editing in human cells. Proc. Natl. Acad. Sci. USA.

[99] Taemaitree L., Shivalingam A., El-Sagheer A.H., Brown T. (2019). An artificial triazole backbone linkage provides a split-and-click strategy to bioactive chemically modified CRISPR sgRNA. Nat. Commun..

[100] Kelley M.L., Strezoska Z., He K.Z., Vermeulen A., Smith A.V. (2016). Versatility of chemically synthesized guide RNAs for CRISPR-Cas9 genome editing. J. Biotechnol..

[101] Ma H.H., Tu L.C., Naseri A., Huisman M., Zhang S.J., Grunwald D., Pederson T. (2016). CRISPR-Cas9 nuclear dynamics and target recognition in living cells. J. Cell Biol..

[102] Hendel A., Bak R.O., Clark J.T., Kennedy A.B., Ryan D.E., Roy S., Steinfeld I., Lunstad B.D., Kaiser R.J., Wilkens A.B. (2015). Chemically modified guide RNAs enhance CRISPR-Cas genome editing in human primary cells. Nat. Biotechnol..

[103] O’Reilly D., Kartje Z.J., Ageely E.A., Malek-Adamian E., Habibian M., Schofield A., Barkau C.L., Rohilla K.J., DeRossett L.B., Weigle A.T. (2019). Extensive CRISPR RNA modification reveals chemical compatibility and structure-activity relationships for Cas9 biochemical activity. Nucleic Acids Res..

[104] Basila M., Kelley M.L., Smith A.V. (2017). Minimal 2′-O-methyl phosphorothioate linkage modification pattern of synthetic guide RNAs for increased stability and efficient CRISPR-Cas9 gene editing avoiding cellular toxicity. PLoS ONE.

[105] Yin H., Song C.Q., Suresh S., Wu Q.Q., Walsh S., Rhym L.H., Mintzer E., Bolukbasi M.F., Zhu L.J., Kauffman K. (2017). Structure-guided chemical modification of guide RNA enables potent non-viral in vivo genome editing. Nat. Biotechnol..

[106] Finn J.D., Smith A.R., Patel M.C., Shaw L., Youniss M.R., van Heteren J., Dirstine T., Ciullo C., Lescarbeau R., Seitzer J. (2018). A Single Administration of CRISPR/Cas9 Lipid Nanoparticles Achieves Robust and Persistent In Vivo Genome Editing. Cell Rep..

[107] Mir A., Alterman J.F., Hassler M.R., Debacker A.J., Hudgens E., Echeverria D., Brodsky M.H., Khvorova A., Watts J.K., Sontheimer E.J. (2018). Heavily and fully modified RNAs guide efficient SpyCas9-mediated genome editing. Nat. Commun..

[108] Ryan D.E., Taussig D., Steinfeld I., Phadnis S.M., Lunstad B.D., Singh M., Vuong X., Okochi K.D., McCaffrey R., Olesiak M. (2018). Improving CRISPR-Cas specificity with chemical modifications in single-guide RNAs. Nucleic Acids Res..

[109] Fu Y., Sander J.D., Reyon D., Cascio V.M., Joung J.K. (2014). Improving CRISPR-Cas nuclease specificity using truncated guide RNAs. Nat. Biotechnol..

[110] Cho S.W., Kim S., Kim Y., Kweon J., Kim H.S., Bae S., Kim J.S. (2014). Analysis of off-target effects of CRISPR/Cas-derived RNA-guided endonucleases and nickases. Genome Res..

[111] Kocak D.D., Josephs E.A., Bhandarkar V., Adkar S.S., Kwon J.B., Gersbach C.A. (2019). Increasing the specificity of CRISPR systems with engineered RNA secondary structures. Nat. Biotechnol..

[112] Cromwell C.R., Sung K., Park J., Krysler A.R., Jovel J., Kim S.K., Hubbard B.P. (2018). Incorporation of bridged nucleic acids into CRISPR RNAs improves Cas9 endonuclease specificity. Nat. Commun..

[113] Rueda F.O., Bista M., Newton M.D., Goeppert A.U., Cuomo M.E., Gordon E., Kroner F., Read J.A., Wrigley J.D., Rueda D. (2017). Mapping the sugar dependency for rational generation of a DNA-RNA hybrid-guided Cas9 endonuclease. Nat. Commun..

[114] Yin H., Song C.Q., Suresh S., Kwan S.Y., Wu Q., Walsh S., Ding J., Bogorad R.L., Zhu L.J., Wolfe S.A. (2018). Partial DNA-guided Cas9 enables genome editing with reduced off-target activity. Nat. Chem. Biol..

[115] Kartje Z.J., Barkau C.L., Rohilla K.J., Ageely E.A., Gagnon K.T. (2018). Chimeric Guides Probe and Enhance Cas9 Biochemical Activity. Biochemistry.

[116] Makarova K.S., Haft D.H., Barrangou R., Brouns S.J., Charpentier E., Horvath P., Moineau S., Mojica F.J., Wolf Y.I., Yakunin A.F. (2011). Evolution and classification of the CRISPR-Cas systems. Nat. Rev. Microbiol..

[117] van der Oost J., Westra E.R., Jackson R.N., Wiedenheft B. (2014). Unravelling the structural and mechanistic basis of CRISPR-Cas systems. Nat. Rev. Microbiol..

[118] Koonin E.V., Makarova K.S. (2019). Origins and evolution of CRISPR-Cas systems. Philos. T R. Soc. B.

[119] Murugan K., Babu K., Sundaresan R., Rajan R., Sashital D.G. (2017). The Revolution Continues: Newly Discovered Systems Expand the CRISPR-Cas Toolkit. Mol. Cell.

[120] Jiang F., Doudna J.A. (2017). CRISPR-Cas9 Structures and Mechanisms. Annu. Rev. Biophys..

[121] Zhu X., Clarke R., Puppala A.K., Chittori S., Merk A., Merrill B.J., Simonovic M., Subramaniam S. (2019). Cryo-EM structures reveal coordinated domain motions that govern DNA cleavage by Cas9. Nat. Struct. Mol. Biol..

[122] Zuo Z.C., Liu J. (2019). Structural and Functional Insights into CRISPR/Cas9 Catalytic Activation and Specificity Enhancement. Biophys. J..

[123] Jinek M., Jiang F.G., Taylor D.W., Sternberg S.H., Kaya E., Ma E.B., Anders C., Hauer M., Zhou K.H., Lin S. (2014). Structures of Cas9 Endonucleases Reveal RNA-Mediated Conformational Activation. Science.

[124] Chen J.S., Dagdas Y.S., Kleinstiver B.P., Welch M.M., Sousa A.A., Harrington L.B., Sternberg S.H., Joung J.K., Yildiz A., Doudna J.A. (2017). Enhanced proofreading governs CRISPR-Cas9 targeting accuracy. Nature.

[125] Kleinstiver B.P., Pattanayak V., Prew M.S., Tsai S.Q., Nguyen N.T., Zheng Z., Joung J.K. (2016). High-fidelity CRISPR-Cas9 nucleases with no detectable genome-wide off-target effects. Nature.

[126] Slaymaker I.M., Gao L., Zetsche B., Scott D.A., Yan W.X., Zhang F. (2016). Rationally engineered Cas9 nucleases with improved specificity. Science.

[127] Casini A., Olivieri M., Petris G., Montagna C., Reginato G., Maule G., Lorenzin F., Prandi D., Romanel A., Demichelis F. (2018). A highly specific SpCas9 variant is identified by in vivo screening in yeast. Nat. Biotechnol..

[128] Hu J.H., Miller S.M., Geurts M.H., Tang W.X., Chen L.W., Sun N., Zeina C.M., Gao X., Rees H.A., Lin Z. (2018). Evolved Cas9 variants with broad PAM compatibility and high DNA specificity. Nature.

[129] Lee J.K., Jeong E., Lee J., Jung M., Shin E., Kim Y.H., Lee K., Jung I., Kim D., Kim S. (2018). Directed evolution of CRISPR-Cas9 to increase its specificity. Nat. Commun..

[130] Vakulskas C., Collingwood M., Behlke M. (2018). High Fidelity Genome Editing with a Novel Mutant HIF1 Cas9. Mol. Ther..

[131] Schmid-Burgk J.L., Gao L.Y., Li D., Gardner Z., Strecker J., Lash B., Zhang F. (2020). Highly Parallel Profiling of Cas9 Variant Specificity. Mol. Cell.

[132] Sledzinski P., Dabrowska M., Nowaczyk M., Olejniczak M. (2021). Paving the way towards precise and safe CRISPR genome editing. Biotechnol. Adv..

[133] Shalem O., Sanjana N.E., Hartenian E., Shi X., Scott D.A., Mikkelsen T.S., Heckl D., Ebert B.L., Root D.E., Doench J.G. (2014). Genome-Scale CRISPR-Cas9 Knockout Screening in Human Cells. Science.

[134] Petkovic I., Bischof J., Kocher T., March O.P., Liemberger B., Hainzl S., Strunk D., Raninger A.M., Binder H.M., Reichelt J. (2022). COL17A1 editing via homology-directed repair in junctional epidermolysis bullosa. Front. Med..

[135] O’Keeffe Ahern J., Lara-Saez I., Zhou D., Murillas R., Bonafont J., Mencia A., Garcia M., Manzanares D., Lynch J., Foley R. (2022). Non-viral delivery of CRISPR-Cas9 complexes for targeted gene editing via a polymer delivery system. Gene Ther..

[136] Neumayer G., Torkelson J.L., Li S., McCarthy K., Zhen H.H., Vangipuram M., Jackow J., Rami A., Hansen C., Guo Z. (2023). A scalable, GMP-compatible, autologous organotypic cell therapy for Dystrophic Epidermolysis Bullosa. bioRxiv.

[137] Lopez-Marquez A., Morin M., Fernandez-Penalver S., Badosa C., Hernandez-Delgado A., Natera-de Benito D., Ortez C., Nascimento A., Grinberg D., Balcells S. (2022). CRISPR/Cas9-Mediated Allele-Specific Disruption of a Dominant COL6A1 Pathogenic Variant Improves Collagen VI Network in Patient Fibroblasts. Int. J. Mol. Sci..

[138] Cong L., Ran F.A., Cox D., Lin S., Barretto R., Habib N., Hsu P.D., Wu X., Jiang W., Marraffini L.A. (2013). Multiplex genome engineering using CRISPR/Cas systems. Science.

[139] Gasiunas G., Barrangou R., Horvath P., Siksnys V. (2012). Cas9-crRNA ribonucleoprotein complex mediates specific DNA cleavage for adaptive immunity in bacteria. Proc. Natl. Acad. Sci. USA.

[140] Dianov G.L., Hubscher U. (2013). Mammalian base excision repair: The forgotten archangel. Nucleic Acids Res..

[141] Ran F.A., Hsu P.D., Lin C.Y., Gootenberg J.S., Konermann S., Trevino A.E., Scott D.A., Inoue A., Matoba S., Zhang Y. (2013). Double Nicking by RNA-Guided CRISPR Cas9 for Enhanced Genome Editing Specificity. Cell.

[142] Kocher T., Wagner R.N., Klausegger A., Guttmann-Gruber C., Hainzl S., Bauer J.W., Reichelt J., Koller U. (2019). Improved Double-Nicking Strategies for COL7A1-Editing by Homologous Recombination. Mol. Ther. Nucleic Acids.

[143] Kocher T., Bischof J., Haas S.A., March O.P., Liemberger B., Hainzl S., Illmer J., Hoog A., Muigg K., Binder H.M. (2021). A non-viral and selection-free COL7A1 HDR approach with improved safety profile for dystrophic epidermolysis bullosa. Mol. Ther. Nucleic Acids.

[144] Bischof J., March O.P., Liemberger B., Haas S.A., Hainzl S., Petkovic I., Leb-Reichl V., Illmer J., Korotchenko E., Klausegger A. (2022). Paired nicking-mediated COL17A1 reframing for junctional epidermolysis bullosa. Mol. Ther..

[145] Hong S.A., Kim S.E., Lee A.Y., Hwang G.H., Kim J.H., Iwata H., Kim S.C., Bae S., Lee S.E. (2022). Therapeutic base editing and prime editing of COL7A1 mutations in recessive dystrophic epidermolysis bullosa. Mol. Ther..

[146] Chen P.J., Hussmann J.A., Yan J., Knipping F., Ravisankar P., Chen P.F., Chen C., Nelson J.W., Newby G.A., Sahin M. (2021). Enhanced prime editing systems by manipulating cellular determinants of editing outcomes. Cell.

[147] Kim H.K., Yu G., Park J., Min S., Lee S., Yoon S., Kim H.H. (2021). Predicting the efficiency of prime editing guide RNAs in human cells. Nat. Biotechnol..

[148] Nelson J.W., Randolph P.B., Shen S.P., Everette K.A., Chen P.J., Anzalone A.V., An M., Newby G.A., Chen J.C., Hsu A. (2022). Engineered pegRNAs improve prime editing efficiency. Nat. Biotechnol..

[149] Doman J.L., Pandey S., Neugebauer M.E., An M., Davis J.R., Randolph P.B., McElroy A., Gao X.D., Raguram A., Richter M.F. (2023). Phage-assisted evolution and protein engineering yield compact, efficient prime editors. Cell.

[150] Yarnall M.T.N., Ioannidi E.I., Schmitt-Ulms C., Krajeski R.N., Lim J., Villiger L., Zhou W.Y., Jiang K.Y., Garushyants S.K., Roberts N. (2023). Drag-and-drop genome insertion of large sequences without double-strand DNA cleavage using CRISPR-directed integrases. Nat. Biotechnol..

[151] Qi L.S., Larson M.H., Gilbert L.A., Doudna J.A., Weissman J.S., Arkin A.P., Lim W.A. (2013). Repurposing CRISPR as an RNA-guided platform for sequence-specific control of gene expression. Cell.

[152] Li Q., Chen J., Minton N.P., Zhang Y., Wen Z., Liu J., Yang H., Zeng Z., Ren X., Yang J. (2016). CRISPR-based genome editing and expression control systems in Clostridium acetobutylicum and Clostridium beijerinckii. Biotechnol. J..

[153] Peters J.M., Colavin A., Shi H., Czarny T.L., Larson M.H., Wong S., Hawkins J.S., Lu C.H.S., Koo B.M., Marta E. (2016). A Comprehensive, CRISPR-based Functional Analysis of Essential Genes in Bacteria. Cell.

[154] Nihongaki Y., Furuhata Y., Otabe T., Hasegawa S., Yoshimoto K., Sato M. (2017). CRISPR-Cas9-based photoactivatable transcription systems to induce neuronal differentiation. Nat. Methods.

[155] Zetsche B., Volz S.E., Zhang F. (2015). A split-Cas9 architecture for inducible genome editing and transcription modulation. Nat. Biotechnol..

[156] Gao Y., Xiong X., Wong S., Charles E.J., Lim W.A., Qi L.S. (2016). Complex transcriptional modulation with orthogonal and inducible dCas9 regulators. Nat. Methods.

[157] Chavez A., Tuttle M., Pruitt B.W., Ewen-Campen B., Chari R., Ter-Ovanesyan D., Haque S.J., Cecchi R.J., Kowal E.J.K., Buchthal J. (2016). Comparison of Cas9 activators in multiple species. Nat. Methods.

[158] Zhou H., Liu J., Zhou C., Gao N., Rao Z., Li H., Hu X., Li C., Yao X., Shen X. (2018). In vivo simultaneous transcriptional activation of multiple genes in the brain using CRISPR-dCas9-activator transgenic mice. Nat. Neurosci..

[159] Gilbert L.A., Larson M.H., Morsut L., Liu Z., Brar G.A., Torres S.E., Stern-Ginossar N., Brandman O., Whitehead E.H., Doudna J.A. (2013). CRISPR-mediated modular RNA-guided regulation of transcription in eukaryotes. Cell.

[160] Gilbert L.A., Horlbeck M.A., Adamson B., Villalta J.E., Chen Y., Whitehead E.H., Guimaraes C., Panning B., Ploegh H.L., Bassik M.C. (2014). Genome-Scale CRISPR-Mediated Control of Gene Repression and Activation. Cell.

[161] Takei Y., Shah S., Harvey S., Qi L.S., Cai L. (2017). Multiplexed Dynamic Imaging of Genomic Loci by Combined CRISPR Imaging and DNA Sequential FISH. Biophys. J..

[162] Ma H.H., Tu L.C., Naseri A., Huisman M., Zhang S.J., Grunwald D., Pederson T. (2016). Multiplexed labeling of genomic loci with dCas9 and engineered sgRNAs using CRISPRainbow. Nat. Biotechnol..

[163] Fu Y., Rocha P.P., Luo V.M., Raviram R., Deng Y., Mazzoni E.O., Skok J.A. (2016). CRISPR-dCas9 and sgRNA scaffolds enable dual-colour live imaging of satellite sequences and repeat-enriched individual loci. Nat. Commun..

[164] Anton T., Bultmann S., Leonhardt H., Markaki Y. (2014). Visualization of specific DNA sequences in living mouse embryonic stem cells with a programmable fluorescent CRISPR/Cas system. Nucleus.

[165] Chen B., Gilbert L.A., Cimini B.A., Schnitzbauer J., Zhang W., Li G.W., Park J., Blackburn E.H., Weissman J.S., Qi L.S. (2013). Dynamic imaging of genomic loci in living human cells by an optimized CRISPR/Cas system. Cell.

[166] Tsai S.Q., Wyvekens N., Khayter C., Foden J.A., Thapar V., Reyon D., Goodwin M.J., Aryee M.J., Joung J.K. (2014). Dimeric CRISPR RNA-guided Fokl nucleases for highly specific genome editing. Nat. Biotechnol..

[167] Guilinger J.P., Thompson D.B., Liu D.R. (2014). Fusion of catalytically inactive Cas9 to FokI nuclease improves the specificity of genome modification. Nat. Biotechnol..

[168] Gaudelli N.M., Komor A.C., Rees H.A., Packer M.S., Badran A.H., Bryson D.I., Liu D.R. (2017). Programmable base editing of A*T to G*C in genomic DNA without DNA cleavage. Nature.

[169] Nishida K., Arazoe T., Yachie N., Banno S., Kakimoto M., Tabata M., Mochizuki M., Miyabe A., Araki M., Hara K.Y. (2016). Targeted nucleotide editing using hybrid prokaryotic and vertebrate adaptive immune systems. Science.

[170] Liu H., Zhu Y., Li M., Gu Z. (2023). Precise genome editing with base editors. Med. Rev..

[171] Osborn M.J., Newby G.A., McElroy A.N., Knipping F., Nielsen S.C., Riddle M.J., Xia L., Chen W., Eide C.R., Webber B.R. (2020). Base Editor Correction of COL7A1 in Recessive Dystrophic Epidermolysis Bullosa Patient-Derived Fibroblasts and iPSCs. J. Investig. Dermatol..

[172] Naso G., Gkazi S.A., Georgiadis C., Jayarajan V., Jackow J., Fleck R., Allison L., Ogunbiyi O.K., McGrath J.A., Ilic D. (2023). Cytosine Deaminase Base Editing to Restore COL7A1 in Dystrophic Epidermolysis Bullosa Human: Murine Skin Model. JID Innov..

[173] Brooks I., Rudnytska M., Gebrezgabher R., Laczmanski L., Newby G., Liu D., McGrath J., Jackow J. (2023). P07 Optimizing DNA specificity and applicability of base and prime editors on COL7A1 variants causing recessive dystrophic epidermolysis bullosa. Br. J. Dermatol..

[174] Porto E.M., Komor A.C. (2023). In the business of base editors: Evolution from bench to bedside. PLoS Biol..

[175] Sledzinski P., Nowaczyk M., Olejniczak M. (2020). Computational Tools and Resources Supporting CRISPR-Cas Experiments. Cells.

[176] Cradick T.J., Qiu P., Lee C.M., Fine E.J., Bao G. (2014). COSMID: A Web-based Tool for Identifying and Validating CRISPR/Cas Off-target Sites. Mol. Ther. Nucleic Acids.

[177] Stemmer M., Thumberger T., Del Sol Keyer M., Wittbrodt J., Mateo J.L. (2015). CCTop: An Intuitive, Flexible and Reliable CRISPR/Cas9 Target Prediction Tool. PLoS ONE.

[178] Bae S., Park J., Kim J.S. (2014). Cas-OFFinder: A fast and versatile algorithm that searches for potential off-target sites of Cas9 RNA-guided endonucleases. Bioinformatics.

[179] Haeussler M., Schönig K., Eckert H., Eschstruth A., Mianné J., Renaud J.B., Schneider-Maunoury S., Shkumatava A., Teboul L., Kent J. (2016). Evaluation of off-target and on-target scoring algorithms and integration into the guide RNA selection tool CRISPOR. Genome Biol..

[180] Moreno-Mateos M.A., Vejnar C.E., Beaudoin J.D., Fernandez J.P., Mis E.K., Khokha M.K., Giraldez A.J. (2015). CRISPRscan: Designing highly efficient sgRNAs for CRISPR-Cas9 targeting in vivo. Nat. Methods.

[181] Labun K., Montague T.G., Gagnon J.A., Thyme S.B., Valen E. (2016). CHOPCHOP v2: A web tool for the next generation of CRISPR genome engineering. Nucleic Acids Res..

[182] Heigwer F., Kerr G., Boutros M. (2014). E-CRISP: Fast CRISPR target site identification. Nat. Methods.

[183] Qiu P., Shandilya H., D’Alessio J.M., O’Connor K., Durocher J., Gerard G.F. (2004). Mutation detection using Surveyor™ nuclease. Biotechniques.

[184] Mashal R.D., Koontz J., Sklar J. (1995). Detection of Mutations by Cleavage of DNA Heteroduplexes with Bacteriophage Resolvases. Nat. Genet..

[185] Brinkman E.K., Chen T., Amendola M., van Steensel B. (2014). Easy quantitative assessment of genome editing by sequence trace decomposition. Nucleic Acids Res..

[186] Bloh K., Kanchana R., Bialk P., Banas K., Zhang Z.G., Yoo B.C., Kmiec E.B. (2021). Deconvolution of Complex DNA Repair (DECODR): Establishing a Novel Deconvolution Algorithm for Comprehensive Analysis of CRISPR-Edited Sanger sequencing Data. Crispr J..

[187] Dehairs J., Talebi A., Cherifi Y., Swinnen J.V. (2016). CRISP-ID: Decoding CRISPR mediated indels by Sanger sequencing. Sci. Rep..

[188] Meienberg J., Bruggmann R., Oexle K., Matyas G. (2016). Clinical sequencing: Is WGS the better WES?. Human. Genet..

[189] Atkins A., Chung C.H., Allen A.G., Dampier W., Gurrola T.E., Sariyer I.K., Nonnemacher M.R., Wigdahl B. (2021). Off-Target Analysis in Gene Editing and Applications for Clinical Translation of CRISPR/Cas9 in HIV-1 Therapy. Front. Genome Ed..

[190] Iyer V., Boroviak K., Thomas M., Doe B., Riva L., Ryder E., Adams D.J. (2018). No unexpected CRISPR-Cas9 off-target activity revealed by trio sequencing of gene-edited mice. PLoS Genet..

[191] Sretenovic S., Green Y., Wu Y., Cheng Y., Zhang T., Van Eck J., Qi Y. (2023). Genome- and transcriptome-wide off-target analyses of a high-efficiency adenine base editor in tomato. Plant Physiol..

[192] Hruscha A., Krawitz P., Rechenberg A., Heinrich V., Hecht J., Haass C., Schmid B. (2013). Efficient CRISPR/Cas9 genome editing with low off-target effects in zebrafish. Development.

[193] Park J., Lim K., Kim J.S., Bae S. (2017). Cas-analyzer: An online tool for assessing genome editing results using NGS data. Bioinformatics.

[194] Clement K., Rees H., Canver M.C., Gehrke J.M., Farouni R., Hsu J.Y., Cole M.A., Liu D.R., Joung J.K., Bauer D.E. (2019). CRISPResso2 provides accurate and rapid genome editing sequence analysis. Nat. Biotechnol..

[195] Kuscu C., Arslan S., Singh R., Thorpe J., Adli M. (2014). Genome-wide analysis reveals characteristics of off-target sites bound by the Cas9 endonuclease. Nat. Biotechnol..

[196] Frock R.L., Hu J., Meyers R.M., Ho Y.J., Kii E., Alt F.W. (2015). Genome-wide detection of DNA double-stranded breaks induced by engineered nucleases. Nat. Biotechnol..

[197] Kim D., Bae S., Park J., Kim E., Kim S., Yu H.R., Hwang J., Kim J.I., Kim J.S. (2015). Digenome-seq: Genome-wide profiling of CRISPR-Cas9 off-target effects in human cells. Nat. Methods.

[198] Tsai S.Q., Zheng Z., Nguyen N.T., Liebers M., Topkar V.V., Thapar V., Wyvekens N., Khayter C., Iafrate A.J., Le L.P. (2015). GUIDE-seq enables genome-wide profiling of off-target cleavage by CRISPR-Cas nucleases. Nat. Biotechnol..

[199] Cameron P., Fuller C.K., Donohoue P.D., Jones B.N., Thompson M.S., Carter M.M., Gradia S., Vidal B., Garner E., Slorach E.M. (2017). Mapping the genomic landscape of CRISPR-Cas9 cleavage. Nat. Methods.

[200] Tsai S.Q., Nguyen N.T., Malagon-Lopez J., Topkar V.V., Aryee M.J., Joung J.K. (2017). CIRCLE-seq: A highly sensitive in vitro screen for genome-wide CRISPR Cas9 nuclease off-targets. Nat. Methods.

[201] Yan W.X., Mirzazadeh R., Garnerone S., Scott D., Schneider M.W., Kallas T., Custodio J., Wernersson E., Li Y.Q., Gao L.Y. (2017). BLISS is a versatile and quantitative method for genome-wide profiling of DNA double-strand breaks. Nat. Commun..

[202] Kim D., Kim J.S. (2018). DIG-seq: A genome-wide CRISPR off-target profiling method using chromatin DNA. Genome Res..

[203] Wienert B., Wyman S.K., Yeh C.D., Conklin B.R., Corn J.E. (2020). CRISPR off-target detection with DISCOVER-seq. Nat. Protoc..

[204] Turchiano G., Andrieux G., Klermund J., Blattner G., Pennucci V., El Gaz M., Monaco G., Poddar S., Mussolino C., Cornu T.I. (2021). Quantitative evaluation of chromosomal rearrangements in gene-edited human stem cells by CAST-Seq. Cell Stem Cell.

